# Tumor Transcriptome Sequencing Reveals Allelic Expression Imbalances Associated with Copy Number Alterations

**DOI:** 10.1371/journal.pone.0009317

**Published:** 2010-02-19

**Authors:** Brian B. Tuch, Rebecca R. Laborde, Xing Xu, Jian Gu, Christina B. Chung, Cinna K. Monighetti, Sarah J. Stanley, Kerry D. Olsen, Jan L. Kasperbauer, Eric J. Moore, Adam J. Broomer, Ruoying Tan, Pius M. Brzoska, Matthew W. Muller, Asim S. Siddiqui, Yan W. Asmann, Yongming Sun, Scott Kuersten, Melissa A. Barker, Francisco M. De La Vega, David I. Smith

**Affiliations:** 1 Life Technologies Inc., Foster City, California, United States of America; 2 Division of Experimental Pathology, Department of Laboratory Medicine and Pathology, Mayo Clinic, Rochester, Minnesota, United States of America; 3 Life Technologies Inc., Austin, Texas, United States of America; 4 Department of Otorhinolaryngology, Mayo Clinic, Rochester, Minnesota, United States of America; 5 Division of Biomedical Statistics and Informatics, Department of Health Sciences Research, Mayo Clinic, Rochester, Minnesota, United States of America; University of California San Diego, United States of America

## Abstract

Due to growing throughput and shrinking cost, massively parallel sequencing is rapidly becoming an attractive alternative to microarrays for the genome-wide study of gene expression and copy number alterations in primary tumors. The sequencing of transcripts (RNA-Seq) should offer several advantages over microarray-based methods, including the ability to detect somatic mutations and accurately measure allele-specific expression. To investigate these advantages we have applied a novel, strand-specific RNA-Seq method to tumors and matched normal tissue from three patients with oral squamous cell carcinomas. Additionally, to better understand the genomic determinants of the gene expression changes observed, we have sequenced the tumor and normal genomes of one of these patients. We demonstrate here that our RNA-Seq method accurately measures allelic imbalance and that measurement on the genome-wide scale yields novel insights into cancer etiology. As expected, the set of genes differentially expressed in the tumors is enriched for cell adhesion and differentiation functions, but, unexpectedly, the set of allelically imbalanced genes is also enriched for these same cancer-related functions. By comparing the transcriptomic perturbations observed in one patient to his underlying normal and tumor genomes, we find that allelic imbalance in the tumor is associated with copy number mutations and that copy number mutations are, in turn, strongly associated with changes in transcript abundance. These results support a model in which allele-specific deletions and duplications drive allele-specific changes in gene expression in the developing tumor.

## Introduction

The development of tools for measuring gene expression and structural variation across the entire genome has revolutionized our ability to characterize cancers at the molecular level. However, such tools have typically relied on microarray hybridization, which has limited sensitivity and is susceptible to the effects of cross-hybridization between homologous DNA fragments. The recent advent of massively parallel sequencing has provided a more powerful tool to study changes in transcriptomes and genomes, through what is termed RNA sequencing (RNA-Seq) [1] and genome re-sequencing [2], respectively. By sequencing the whole transcriptomes of a tumor and matched normal tissue, we can compare not only the relative abundance of annotated transcripts, but also that of non-annotated transcripts, transcript isoforms and different alleles [3], [4], [5]. With sufficient sequencing depth, tumor-specific mutations, which may contribute to pathogenesis, can be detected. Similarly, by sequencing the genomes of a tumor and matched normal tissue, structural and point mutations associated with tumor development can be discovered [6], [7], [8], [9].

Cancers of the head and neck are the sixth most commonly observed cancers worldwide [10]. Most are squamous cell carcinomas that commonly occur in the oropharynx and oral cavity. At the advanced stage, these cancers are highly invasive and metastatic, with an associated five year survival in the United States of only 50% [11]. Microarray studies of oral cavity tumors have revealed genes that are consistently mis-expressed [12], [13], [14], [15], [16], but have not yet led to a panel of genes that can be used effectively to make informed clinical decisions.

Here we have paired a new strand-specific whole transcriptome library preparation method with massively parallel ligation sequencing to study the transcriptomes of three oral squamous cell carcinoma (OSCC) tumors and three matched normal tissues. With the resulting 60 Gb of sequence we performed two types of analyses. First, we examined differential expression of genes between tumor and normal tissue across the three patients and compared these results to those produced by microarray and RT-qPCR. The comparison reveals strong concordance between the methods, with RNA-Seq outperforming microarrays at measurement of the low abundance transcripts. Second, we investigated the extent and types of allelic imbalance (AI) observed between the tumor and normal tissues of the three patients. Here we focus on relative AI, which compares the ratio of the expression of two alleles in one sample (e.g., tumor tissue) to that in another sample (e.g., matched normal tissue). AI represents a convolution of genotype and expression level that can arise due to a number of different processes. Our analysis demonstrates the ability of RNA-Seq to accurately measure AI and the utility of AI for understanding cancer development. Unlike other methods, our RNA-Seq approach surveys strand-specific expression across the entire length of transcripts, allowing us to observe bidirectional promoter usage and improving our chances of covering the rare heterozygous SNPs needed for AI analysis.

We have also sequenced the tumor and normal genomes of one of the three patients and determined copy number changes present in the tumor genome. By comparing genomic and transcriptomic data from this patient, we observe that changes in gene dosage are strongly associated with changes in gene expression and allelic imbalance in this tumor. These data are consistent with a model in which allele-specific duplication and deletion drive allele-specific changes in gene expression [17], [18], [19].

## Results

### Measurement of Gene Expression in OSCC and Normal Tissue by RNA-Seq

**Figure 1:**
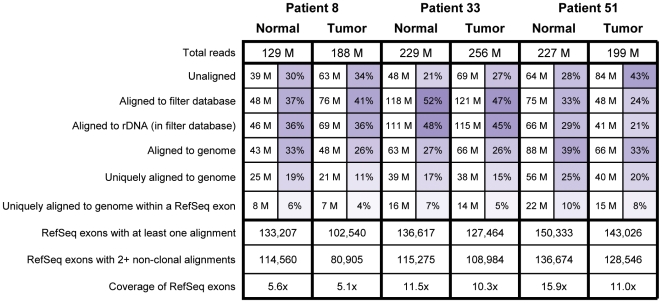
Alignment statistics for transcriptome reads from the six clinical samples. Read counts listed in the middle section are expressed in millions (left column) or as a percentage of the total reads processed (right column) for each sample.

We sequenced rRNA-depleted total RNA extracted from tumor and matched normal tissue from three patients with oral squamous cell carcinoma (OSCC) (Methods and Figure S1), yielding 129–256 million 50 bp long sequence reads per sample. Reads were aligned first to a filter database (containing, e.g., rDNA sequences) and then to the human genome (Methods and Figure S2) and an overview of the results is displayed in Figure 1. The majority of reads from each sample (57–79%) were successfully aligned; those that did not align are likely to be polyclonal, low quality or have origins outside the reference human genome. A large fraction of the reads from each sample (21–48%) aligned to rDNA, suggesting that our rRNA depletion efficiency was low, which is most likely because the RNA isolated from these tumors was partially degraded (Figure S3). Nevertheless, between 21 and 56 million reads per sample were uniquely aligned to the human genome outside of rDNA and over 100,000 of the 195,148 RefSeq-annotated exons were detected in each sample (Figure 1 and Text S1). On the whole, we have an unprecedented depth of uniquely aligned sequence from these tumor and matched-normal RNA samples.

Given that RNA-Seq is still an emerging methodology, we wanted to ensure that our method provides gene expression measurements that are consistent with orthogonal technologies (e.g., RT-qPCR and microarray). We assessed concordance with these other methods at the level of differential gene expression between tumor and normal tissue samples (Methods). The same six RNA samples used for RNA-Seq were prepared and hybridized to microarrays that have one probe designed to target each of the RefSeq transcripts (Methods). The two gene expression platforms are strongly concordant in the measurement of differential gene expression between tumor and normal tissue (ρ = 0.73-0.81; Figure S4 and Tables S1 and S2). However, it is apparent that the two platforms diverge at low expression levels, where RNA-Seq often indicates differential expression and the microarray does not. We randomly selected sixteen of these divergent genes for validation by RT-qPCR, eight that were up-regulated and eight that were down-regulated in the tumor relative to normal tissue, and found that the measurements provided by RT-qPCR are more highly correlated to those provided by RNA-Seq (ρ = 0.84), than to those provided by microarray (ρ = 0.71) (Methods and Figure S4e,f). More strikingly, the slope of the regression line for the RNA-Seq comparison is 1.0, while that for the microarray comparison is 0.15, suggesting that the fold-changes measured by microarray for these genes are strongly compressed relative to the true fold-changes and helping to explain the observed discrepancies between the RNA-Seq and microarray data. These results demonstrate that our RNA-Seq method can reliably measure gene expression differences in human tissue and further suggest that RNA-Seq is better at making such measurements when expression levels are low.

### A Common Set of Genes Is Differentially Expressed in OSCC

**Figure 2:**
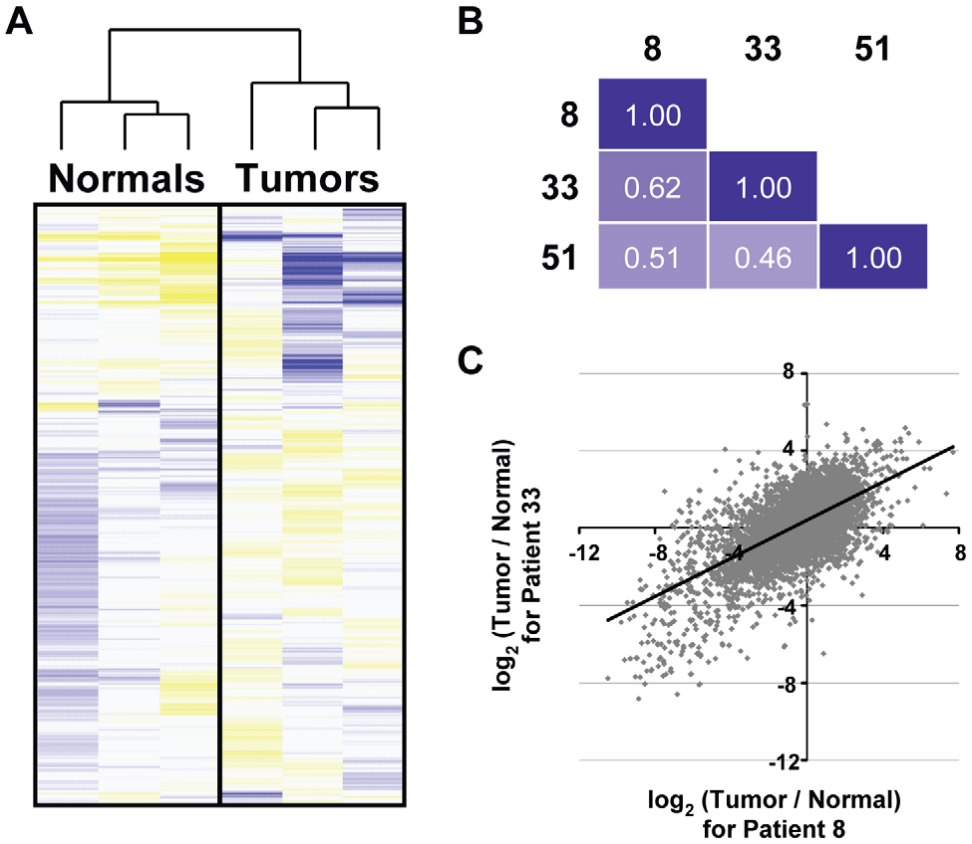
A common set of genes is differentially expressed in the tumors of three patients with oral carcinoma. (A) Transcript expression levels in each of the six samples were hierarchically clustered and, as expected, the normal and tumor tissues form tight clusters. Shades of blue indicate lowered expression, relative to the mean across samples, whereas shades of yellow indicate higher expression relative to the mean. (B) For each patient, gene expression in the tumor was compared to that in matched normal tissue. Pearson correlations indicate strong and significant (P<10^−16^) similarity of differential transcript expression across the three patients. (C) A scatterplot, comparing differential transcript expression between patients 8 and 33.

We next investigated the biological significance of the tumor versus normal (TvN) expression profiles. First, by hierarchically clustering the log-transformed transcript expression levels in the six samples, we established that the same types of tissue from different patients are more similar than different types of tissue from the same patient (Figure 2a). Furthermore, we observed strong correlations (ρ = 0.46–0.62) between the log-transformed TvN expression profiles between patients (Figure 2b), confirming that there is a common gene expression perturbation associated with this disease in different individuals. Close examination of the relationship between the TvN profiles of different patients (Figure 2c), revealed that the correlation extends across the entire range of fold-changes (e.g., ρ = 0.36 for genes with | log_2_ T/N |<2.0).

**Figure 3:**
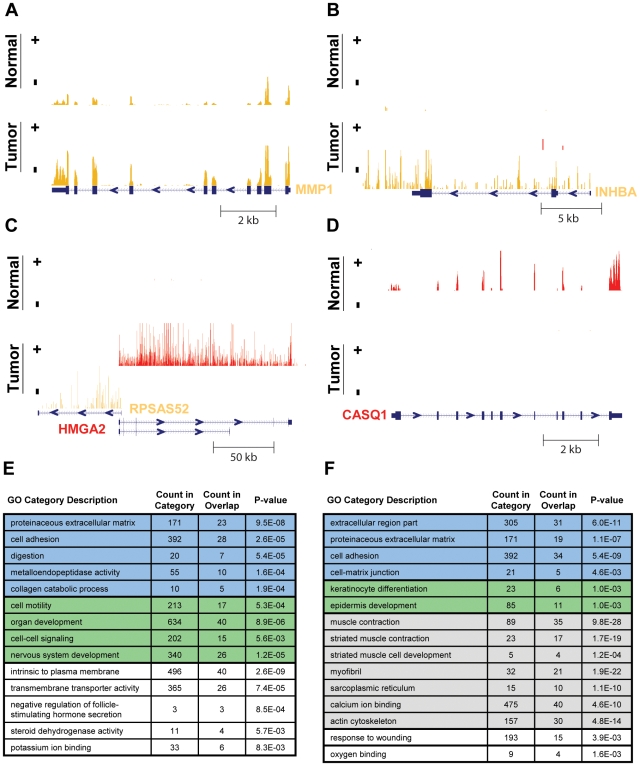
The genes commonly mis-regulated across the three cases of oral carcinoma function in cell differentiation, adhesion, extracellular matrix digestion and muscle contraction. (A–D) Examples of gene expression at four loci. Plotted across each locus is the normalized sequence coverage on both the plus (colored red) and minus (colored orange) strands, for the tumor and normal tissue of a particular patient. (A) MMP1 in patient 51; y-axis scale is 10 to 2000. (B) INHBA in patient 8; y-axis scale is 10 to 150. (C) HMGA2 and RPSAS52 in patient 8; y-axis scale is 10 to 300 for the plus strand and 10 to 100 for the minus strand. (D) CASQ1 in patient 33; y-axis scale is 10 to 100. (E–F) The most up-regulated (E) and down-regulated (F) genes in the tumors of the three patients were submitted for gene ontology (GO) analysis [29] to identify biological processes and components that are typically mis-regulated in oral cancer. Redundant GO categories were filtered. “Count in category” indicates the total number of genes assigned to a given GO category and “count in overlap” indicates the number of up- or down- regulated genes that are also assigned to the given GO category.

To isolate the set of genes commonly mis-regulated in the development of OSCC, we took a rank-order approach (Methods and Tables S1 and Tables S3). We note that an approach employing a likelihood ratio test [20] yielded similar results (Table S1) that do not alter any of the conclusion made here. A survey of the genes most strongly differentially expressed across the three patients, reveals four particularly interesting genes (Figure 3a–d): (1) MMP1, which encodes a secreted matrix metalloproteinase that breaks down interstitial collagens, ranks third amongst the set of up-regulated genes. MMP1 is part of a family of matrix metalloproteinases with known roles in invasion and metastasis [21] and several family members are amongst our most up-regulated genes (MMP3/10/11/12/15). Unlike microarrays, which typically measure gene expression in only one or a few regions of a gene specifically targeted by probes, our RNA-Seq method measures strand-specific expression across the entire gene, as can clearly be seen for MMP1 (Figure 3a). (2) INHBA, the fourth most up-regulated gene across the patients, similarly shows strand-specific expression spanning its locus (Figure 3b). INHBA encodes the beta A subunit that, together with an alpha subunit encoded by INHA, forms inhibin. Inhibin is a pituitary FSH secretion inhibitor that has been shown to regulate gonadal stromal cell proliferation negatively and to have tumor-suppressor activity [22]. However, INHBA's increased expression levels in these patients' tumors more likely reflect its product's ability to homodimerize and form activin A, an FSH secretion activator and putative oncogene in esophageal carcinoma [23]. This is consistent with the apparent lack of expression of INHA in the normal and tumor tissues of all three patients and implicates INHBA as a potential oncogene in OSCC. (3) HMGA2 is the eleventh most up-regulated gene across the patients and is of considerable interest as a known oncogene [24], [25], [26], containing an HMG DNA-binding domain. HMGA2 was not frequently featured in previous microarray studies of OSCC, possibly due to the low overall expression of its exons (HMGA2's expression, as judged by transcript length-normalized read counts, ranks in the bottom 2% of all transcripts). In our microarray experiments, we find only very modest TvN changes in HMGA2's expression (0.9-, 1.1- and 1.5-fold), compared to those changes measured by RNA-Seq (2.0-, 15.2- and 206-fold). However, as Figure 3c clearly illustrates, HMGA2's exonic and intronic regions are strongly up-regulated in the tumor. Furthermore, we validated HMGA2's differential expression by RT-qPCR (Figure S4e, discussed in the previous section). Interestingly, the up-regulation of HMGA2 coincides with the up-regulation of a pseudogene for ribosomal protein SA (RPSAP52), whose expression runs in the opposite direction and overlaps HMGA2 by roughly 2 kb in patient 8, suggesting that these two genes share a bi-directional promoter [27], [28]. (4) CASQ1, the sixth most down-regulated gene across the patients (Figure 3d), encodes calsequestrin, a calcium binding glycoprotein that localizes to the sarcoplasmic reticulum and may act to store calcium. The functions of CASQ1 and the three other genes are emblematic of the functions encoded by the entire set of differentially expressed genes.

To more systematically assess the biological functions of commonly mis-regulated genes, we performed gene ontology (GO) analysis [29]. A selection of the results is displayed in Figure 3e,f. The process of cell adhesion and components of the extra-cellular matrix (ECM) feature prominently amongst both the up- and down-regulated gene sets (shaded blue in Figure 3e,f), of which MMP1 is a prime example (Figure 3a). The altered regulation of these genes is consistent with the model that cancer development involves restructuring of the external environment, including breakdown of the ECM [30], [31], and is also consistent with previous microarray studies of oral cancer (e.g., [32]). Also featured in both gene sets are functions related to development and differentiation (shaded green in Figure 3e,f). Whereas the down-regulated genes tend to function in epidermal development, the up-regulated genes more often function in cell motility and nervous system development, as might be expected for OSCC [33], [34]. Perhaps the strongest trend though, is the massive over-representation of muscle contraction functions amongst the down-regulated genes (shaded gray in Figure 3f). Several actins, myosins and related components of the cytoskeleton are strongly down-regulated, possibly reflecting a de-differentiation of the muscle phenotype during oral cancer development. Alternatively, this may simply reflect the choice of normal tongue tissue as our control. Alterations to cell adhesion, development, differentiation, and the ECM are consistent with what is known about this disease.

The results presented here are also consistent with existing knowledge of gene expression changes in OSCC development. For example, a meta-analysis of 41 head and neck squamous cell carcinoma (HNSCC; OSCC is one type of HNSCC) expression profiling studies identified 25 genes that were differentially expressed between tumor and normal tissue in nine or more of these studies [16]. Of these 25 genes, 15 are also in our set of 600 mis-regulated genes, which is much greater than the roughly 1.4 genes expected by chance (hypergeometric *P*-value<10^−12^; 15 of 25, from 10,542 total genes detected) and also much greater than the mean of 6.8 genes for any given study in the meta-analysis. Thus, it is evident that the examination of a relatively small number of OSCC cases by RNA-Seq recapitulates much of the accumulated knowledge of gene expression alterations in the development of this cancer, including the mis-regulation of genes functioning in cell adhesion, development, differentiation, muscle contraction and the ECM.

### There Is Widespread Allelic Imbalance between Tumor and Normal Tissues

**Figure 4:**
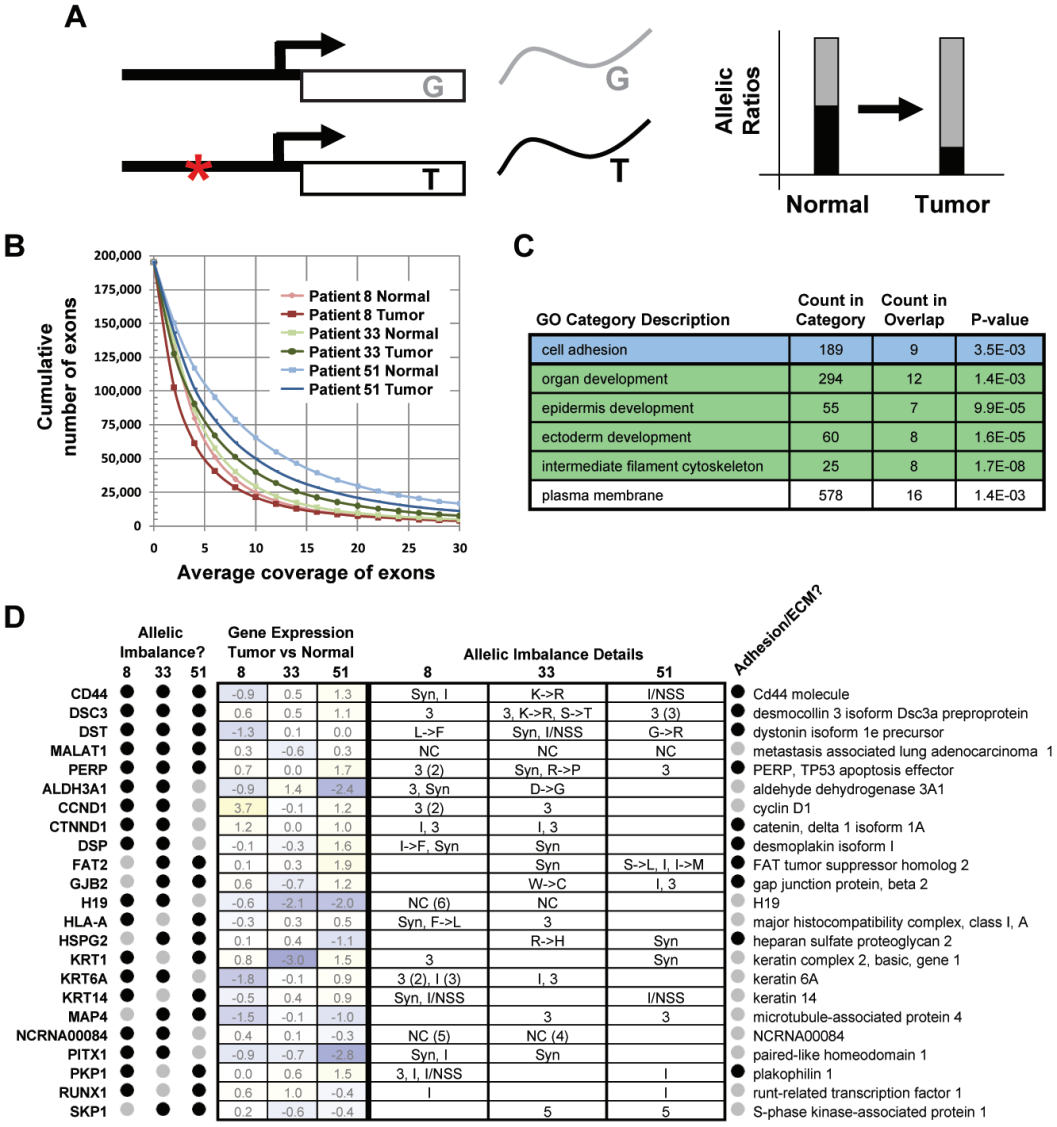
A common set of genes, functioning in cell adhesion and development, exhibits allelic imbalance in the tumor transcriptomes of three patients with oral carcinoma. Allelic imbalance (AI) at the RNA level can arise in a number of ways, including through point mutation and changes in the relative expression of alleles (aka, allele-specific expression). (A) Illustrated is an example of two pre-existing alleles (G and T), one of which undergoes a linked promoter mutation (red asterisk) in the tumor, relative to the normal tissue. If, for example, the mutation alters a *cis*-regulatory element, then the balance of the two alelles (G and T) may change. (B) In principle, there is enough sequence coverage to discover AI for a large number of exons. For example, more than 25,000 exons have at least 10x coverage (averaged across all sites in the exon). (C) Genes with one or more instances of allelic imbalance in the tumors of two or more patients were submitted for GO analysis [29] to identify biological processes and components that are typically allelically imbalanced in oral cancer. Redundant GO categories were filtered. (D) A selection of genes with allelic imbalance in two or more patients is listed, along with the log_2_ differential expression of each gene and regions of gene structure impacted. Also noted is whether or not the gene is involved in cell adhesion or is a component of the ECM. Key: 3 = 3′ UTR, I = Intronic, NSS = Near Splice Site, Syn = Synonymous, → = Non-Synonymous.

One advantage of our RNA-Seq method is that it allows us to address questions that were previously inaccessible on the genome-wide scale. An example is allelic imbalance (AI), which we define here as a difference in the nucleotide frequencies at a given transcriptome position between two tissues/conditions (Figure 4a). AI can arise through one, or a combination of, several processes acting in one tissue relative to the other: (1) *cis* mutations that impact the underlying genomic position directly in one of the two haplotypes (e.g., a point mutation or a deletion/duplication that changes the copy number of one allele relative to the other), (2) indirect *cis* mutations (e.g., a point mutation in an upstream *cis*-regulatory element affecting expression of only that allele), (3) differences in *trans* (e.g., mutations to other components of the transcriptional network that differently affect expression of the alleles), (4) differences in RNA editing. More simply, AI represents a convolution of genotype and expression level. Here we demonstrate that RNA-Seq can be employed to successfully interrogate AI in tumors, relative to matched normal tissue, across the whole transcriptome. Although, in general, we will not be able to differentiate between the four possible underlying causes from RNA-Seq data alone, we show here that the presence of AI itself is informative and that straightforward follow-up experiments can be utilized to identify the root cause.

In principle, our depth of sequencing is sufficient to detect AI in a large number of exons (Figure 4b); the average per base exon coverage varies between 5x and 16x, depending on the sample (Figure 1). However, it is unknown whether the nucleotide counts accurately reflect the underlying expression of each allele. To ensure this is the case, we examined the distribution of allelic ratios. For a given genomic coordinate, the “allelic ratio” is the number of reads aligned across that position that indicate the reference nucleotide divided by the number of reads that indicate the first non-reference nucleotide in dbSNP [35]. Thus we only concern ourselves here with the subset of genomic positions for which an allele is listed in dbSNP and to which at least 15 reads are aligned. If alleles tend to be expressed at equal levels, then we expect to see a trimodal distribution of allelic ratios, representing the three possible genotypes: homozygous reference, heterozygous and homozygous non-reference. However, if our technology does not accurately quantify the two alleles or if alleles tend to vary greatly in their expression, then we would expect to see a more uniform distribution. In fact, clear trimodal distributions are observed in all six samples (Figure S5) with peaks likely representing the three genotypes. Thus we are confident that the nucleotide counts accurately portray the expression levels of the different alleles.

We next developed a method to detect relative AI between samples (Methods) and applied it to the three paired samples. Here we analyze further only those genomic positions having, (1) at least 15x coverage in the tumor and normal samples, (2) significant AI with χ^2^ *P*-value less than 10^−3^ (Figure S5), and (3) not more than 90% of covering reads supporting each nucleotide aligned to identical positions in the genome (see Methods for full explanation). Presence in dbSNP is not a requirement, thus in theory we can detect imbalances due to either *de novo* point mutation or altered expression of preexisting variants. 1316, 991, and 3221 genomic positions meet these criteria in patients 8, 33, and 51, respectively, demonstrating that widespread allelic imbalance is associated with the development of these tumors. In what follows, we further narrowed our focus to those positions with a greater than 10% change in the absolute frequency of at least one nucleotide between the two samples, surmising that these positions would be of greatest biological interest. After applying this filter, we are left with 229, 185, and 345 genomic positions from 121, 134, and 209 unique genes in patients 8, 33, and 51, respectively (Table S4). The sets of genes allelically imbalanced in the three patients overlap significantly; 20 to 26 genes overlap by pair-wise comparison (hypergeometric *P*-values ranging from 10^−7^ to 10^−11^) and seven genes are common to all three patients.

To assess whether or not the allelically imbalanced genes are biologically meaningful and related to the development of this cancer, we performed GO analysis[29]. Interestingly, the functions enriched in the set of 52 genes allelically imbalanced in more than one patient (Table S5) are largely the same as those enriched in the set of differentially expressed genes (Figure 4c; compare to Figure 3e,f). Despite this functional overlap, the actual genes in these two sets overlap very little (only 4 of these 52 genes are also in the list of commonly mis-regulated genes from the previous section; 8% observed versus 12% expected, *P*-value = 0.88), indicating that allelic imbalance may offer a new avenue for discovering genes involved in cancer development. This lack of significant overlap between AI genes and differentially expressed genes holds true even when the cutoffs used to define the set of differentially expressed genes are varied substantially (not shown), which indicates that it would not be possible to detect the AI genes from differential gene expression data alone. Nevertheless the functions of these genes indicate they are probably of relevance to tumorigenesis. To test this further we employed the NextBio Professional system [36] to search for studies and diseases in which the set of AI genes were differentially expressed. We found that most of the top hits were from cancer-related studies and diseases, such as melanoma [37], follicular lymphoma [38], and squamous cell lung cancer [39], further demonstrating the relevance of AI to studies of cancer development.

Of these 52 genes with AI in two or more patients, 23 appeared particularly interesting and are highlighted in Figure 4d. Among these 23 we detect two cases of apparent non-synonymous point mutations (within DST and GJB2), where only one nucleotide was sequenced in the normal tissue and two were sequenced in the tumor (and the second is not present in dbSNP). Similarly, we observe one case of point mutation in the 3′ UTR of a gene (for KRT1) and a couple cases of apparent point mutations within 10 bp of a splice site (for DST and S100A2). Changes that apparently reflect a loss of heterozygosity or complete silencing of one allele in the tumor are even more frequent. In several cases, these result in higher expression of an allele that has a non-synonymous or 3′ UTR mutation relative to the other allele (e.g., DST, CCND1, DSP, PKP1, KRT6A, ALDH3A1, DSC3, FAT2 and GJB2). We have performed extensive validation of our AI results as described in the Methods section and depicted in Figure S6. Among those genes with validated AI is DST, a member of the plakin protein family of adhesion junction plaque proteins. While the expression of DST in tumor versus normal changes only very modestly across patients (less than 2-fold), DST is among the small set of genes that are allelically imbalanced in all three patients. Furthermore, DST has been shown to be perturbed in studies of other cancers, such as squamous cell carcinoma of the lung [40], making it an interesting target of further study.

**Figure 5:**
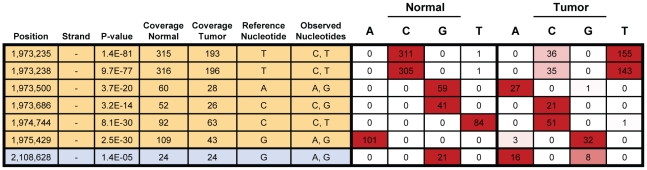
A switch in the genomic imprinting of H19 and IGF2. There are six sites in H19 (shaded orange) and one in IGF2 (shaded blue) on chromosome 11 that are apparently heterozygous in patient 8 (five were validated by as-qPCR). In normal tissue, most detectable expression of H19 is from one allele, as expected for this imprinted, maternally expressed gene. Unexpectedly, in the tumor, nearly all detected expression is from the other, presumably paternal, allele. Observed nucleotides are from dbSNP[35].

One particularly convincing example of AI is observed for transcripts of the adjacent IGF2 and H19 genes (Figure 5), which are implicated in carcinogenesis and encode an insulin-like growth factor and a non-coding RNA, respectively [41], [42], [43], [44]. There are seven genomic positions in these two genes that are allelically imbalanced in patient 8. The seven positions are located within a 135 kb window on chromosome 11 and all are polymorphic in the human population, increasing the likelihood that patient 8 may be heterozygous at these sites. All seven sites show a striking pattern: while one nucleotide is sequenced in the normal tissue, it is primarily another nucleotide that is sequenced in the tumor tissue. We tested and verified that patient 8 is indeed heterozygous at five of these seven sites by allele-specific qPCR (as-qPCR) of normal and tumor genomic DNA (the other two sites were not tested; Methods and Figure S6a). We also validated the observed changes in allele-specific expression by allele-specific reverse transcription qPCR (as-RT-qPCR) of normal and tumor cDNA (Figure S6a). Interestingly, H19 and IGF2 are imprinted in the germline by methylation and are normally only expressed in somatic tissues from the maternal and paternal alleles, respectively [43], [45]. Thus, the simplest explanation for these observations is that there was mono-allelic expression of H19/IGF2 in the normal tissue, which was subsequently lost or allelically switched during tumor development. Loss of imprinting is frequently observed in the development of some cancers [42], [46], [47], [48] and can lead to tumorigenesis [49]. However, this is the first time, to our knowledge, that expression has been observed to flip from one allele to the other.

In summary, our whole transcriptome sequencing approach allows detection of AI across a large number of genes. AI is a widespread phenomenon in oral cancer development, impacting genes known or likely to be involved in cancer etiology. AI's presence can be used effectively to identify key cancer development events, such as non-synonymous point mutations, loss of imprinting, loss of heterozygosity, and copy number changes resulting in up-regulation of one allele relative to the other. However, resolving ambiguity between these possible underlying causes currently requires follow-up experiments.

### Structural Variation Underlies the Gene Expression Changes Observed in One Patient

There is abundant copy number variation (CNV) in mammalian populations [50], [51], which can sometimes be associated with changes in the expression of overlapping and nearby genes [52], [53] and also with human traits and diseases [54], [55]. We sought to understand whether copy number (CN) mutations in OSCC development were driving the changes in gene expression we observed. We therefore sequenced the tumor and normal genomes of patient 8, to obtain sequence coverage of ∼0.8x (Methods). We applied a recently developed CNV segmentation algorithm [56], designed for paired-sample next-generation sequence data, to the resulting read sequence alignments (Methods). This produced 328 genomic segments, with TvN copy number changes (CNCs) ranging from 0.4 to 8.8 (Table S6). An array comparative genomic hybridization (aCGH) experiment was performed on the same tumor sample, resulting in highly concordant CN segments (Methods and Figure S7b). CNCs were also validated at 23 particularly interesting genes by qPCR on the samples of patient 8 and across a panel of OSCC tumors obtained from 13 additional patients. For patient 8, 22 of 23 CNCs were successfully validated (Figure S7a). Across the panel of other patients, CNCs at these 23 genes ranged in prevalence from 7% to 43%, with a mean of 24% (Figure S7c).

**Figure 6:**
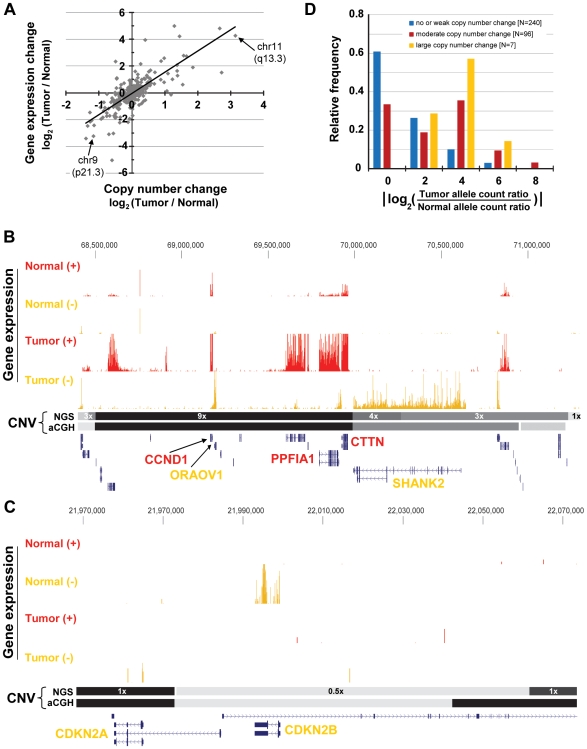
Large structural mutations are strongly correlated with the changes in gene expression observed in one patient's tumor. (A) A strong correlation (ρ = 0.73) is observed between changes in copy number and changes in gene expression for patient 8. The correlation is stronger (ρ = 0.84) if only copy number changes greater than 1.4-fold are considered. (B) The most strongly amplified region (9-fold more copies in the tumor than normal; chr11:68,503,204-69,987,273) contains several differentially expressed (red and orange tracks) genes, highlighted in the text. (C) One region (chr9:21,973,361-22,061,522) that is likely to have been deleted in the tumor contains two genes of interest: cyclin-dependent kinase inhibitor 2B (CDKN2B) and cyclin-dependent kinase inhibitor 2A (CDKN2A). (D) Given that gene dosage changes are strongly associated with gene expression changes, it is expected that heterozygous amplifications and deletions will be associated with the allelic imbalance of transcripts. Shown are the distributions of allelic imbalance for genomic regions that fall into one of three categories of log-transformed copy number change (CNC): low or no CNC (blue; |CNC|<1.2), moderate CNC (red; 1.2<|CNC|<1.8), and large CNC (yellow; |CNC|>1.8). The moderate and large CNC distributions are shifted to significantly higher values of allelic imbalance compared to the no CNC distribution (Mann-Whitney P-values of 10^−10^ and 10^−3^, respectively).

To compare the CNCs observed between normal and tumor tissue to changes in gene expression, we calculated the differential expression of each genomic segment (Methods). The log_2_-transformed CNC and log_2_-transformed normalized gene expression ratio for each CN segment were then plotted (Figure 6a), revealing a strong relationship between CNCs and changes in gene expression (ρ = 0.73).

One particularly remarkable example of concordant change in CN and gene expression involves the 9x amplification of a 1.5 Mb segment of chromosome 11 (Figure 6b). Two immediately adjacent segments, roughly 0.3 Mb and 1.0 Mb in length, are also amplified (4x and 3x, respectively), suggesting that multiple, nested duplications gave rise to the amplified state observed in the tumor. Contained within the three amplified regions are ∼20 genes, several of which have been highlighted for their functional relevance and frequent amplification in OSCCs [57]: Cyclin D1 (CCND1) is an oncogene previously implicated in several cancers [58] and is 13-fold up-regulated in the tumor. Cortactin (CTTN), which is often over-expressed in HNSCCs [59] and helps organize the cytoskeleton and cell adhesion structures of epithelia and carcinoma cells, is 14-fold up-regulated. Finally, SH3 and multiple ankyrin repeat domains 2 (SHANK2), which binds the SH3 domain of cortactin and is thereby thought to promote cell motility at growth cones of neurons [60], is 51-fold up-regulated in the tumor. Copy number changes in CTTN and SHANK2 were strongly and equally prevalent across our panel of 14 patients assayed by qPCR (43%; Figure S7c).

Another striking example of concordant copy number and gene expression change occurs on chromosome 9, where a 90 kb genomic segment, containing two genes, has apparently been deleted in the tumor (Figure 6c). One gene, cyclin-dependent kinase inhibitor 2B (CDKN2B), falls completely within the deleted region. CDKN2B controls G1 cell cycle progression by inhibiting Cdk4 or Cdk6 [61]. Its cell growth regulation function and 120-fold down-regulation in the tumor make it a strong candidate for a tumor suppressor [62]. Copy number changes at CDKNB were prevalent across our panel of 14 patients (29%; Figure S7c). CDKN2B's neighbor, cyclin-dependent kinase inhibitor 2A (CDKN2A), functions similarly to inhibit Cdk4 and is a known tumor suppressor [63], but is not detectably expressed in either normal or tumor tissue.

It is also worth noting the amplification of Wnt inhibitory factor 1 (WIF1), which encodes an extracellular component of the Wnt pathway that plays a critical role in regulating cell adhesion, proliferation and differentiation [64], [65]. Despite its amplification, WIF1's expression is strongly down-regulated across the three tumors for which RNA was sequenced, demonstrating that amplification does not always imply increased expression. Down-regulation of WIF1 has been reported in prostate, breast, lung, bladder, salivary gland and esophageal cancers [66], [67] and evidence of promoter hypermethylation in Barrett's esophagus cases that progress to esophageal adenocarcinoma suggests that loss of expression likely represents an early event in carcinogenesis [66]. Copy number change at WIF1 was 36% prevalent across the 14 tumors for which we measured CNC, but interestingly the direction of change was not consistent: two of the tumors had WIF1 amplifications and three others had deletions. This suggests that the down regulation of WIF1 can arise not only from epigenetic alterations, but also from deletion and rearrangement events and that loss of WIF1 expression may be involved with carcinogenesis of OSCC.

Finally, we examined the relationship between allelic imbalance and copy number change. Given that gene dosage changes are strongly associated with gene expression changes, it is expected that heterozygous amplifications and deletions will be associated with the allelic imbalance of transcripts. For example, if one allele of a genomic region is amplified 10-fold relative to the other allele, then we might expect to see a 10-fold imbalance of heterozygous SNPs that fall within the region (depending on the exact mechanism of amplification). We grouped each copy number segment into one of three log_2_-transformed CNC categories, disregarding the direction of change: low or no CNC (|CNC|<1.2), moderate CNC (1.2<|CNC|<1.8), and large CNC (|CNC|>1.8). We then considered the distributions of AI for genomic regions in each of those CNC categories (Figure 6d). Here we consider only genomic positions, (1) with at least one allele in dbSNP, and (2) at which the two nucleotides (reference and first dbSBP allele) are present in at least four reads in the normal tissue sample. These two criteria ensure that the patient is very likely to be heterozygous at such positions in his normal tissue. Because we do not know *a priori* which allele has changed in copy number, we consider only the absolute value of AI. As depicted in Figure 6d, the moderate and large CNC categories are significantly shifted to higher levels of AI, relative to the low/no CNC category (Mann-Whitney *P*-values 10^−10^ and 10^−3^, respectively). Thus, CNC is associated with increased AI in this patient's tumor. For example, the region of chromosome 11 mentioned above for its 9-fold amplification in the tumor, harbors two expressed heterozygous SNPs within one gene, CCND1. As expected if the region is amplified in one of the two homologous chromosomes and not the other, the sites are more than 16-fold imbalanced in the tumor compared to the normal tissue, a result which we validated by as-qPCR of genomic DNA and cDNA (Figure S6).

Taken together, these results show a strong relationship between structural mutations and changes in gene expression in this patient's tumor. Increased gene dosage is associated with increased gene expression and decreased gene dosage is typically associated with decreased gene expression. Furthermore, gene dosage changes are linked with changes in the relative expression of alleles. The simplest interpretation is that the allele-specific structural mutations in this patient's tumor have driven the observed changes in gene expression.

## Discussion

By sequencing the tumor and normal transcriptomes of three individuals with OSCC, we have characterized, in depth, the changes in gene expression associated with development of this cancer. We have demonstrated that our RNA-Seq method can be successfully applied to profile gene expression in tumor and matched normal tissues, in much the same way that microarrays have been applied to this task in the past. The gene expression profiling results we produced by sequencing are largely concordant with those we produced from hybridizing the same samples to microarrays (Figure S4). However, as we have shown here by RT-qPCR, sequencing is a superior approach for measuring differential expression of genes expressed at low levels, at least for samples of imperfect quality, such as the clinical tissue samples studied here. For example, the 206-fold up-regulation of the transcriptional regulator and oncogene HMGA2 in one tumor would have been missed if only assayed by microarray (Figure 3c and Figure S4e). In general, we expect that deep sequencing methods will better detect perturbations in the expression of genes encoding transcription factors and signaling proteins, which tend to be expressed at lower levels than other genes in the cell [68]. HMGA2 also has a transcriptional coverage profile that indicates it shares a bi-directional promoter with RPSAP52 (Figure 3c), neatly demonstrating the added insights possible with a strand-specific, whole transcriptome approach to RNA-Seq.

Not only have we demonstrated the overall similarity of results obtained by deep sequencing and microarray hybridization of the same samples, but we have also shown that both sets of results are in strong agreement with existing knowledge of this cancer. Development of OSCC involves perturbed regulation of genes functioning in interaction with the external environment, such as those functioning in cell adhesion and encoding components of the extra-cellular matrix (Figure 3a,e,f). Our functional analysis also indicates extensive down-regulation, in the tumors, of genes functioning in epidermal development and up-regulation of genes functioning in cell motility. The strongest gene expression signature observed is the down-regulation in tumors of many genes with muscle contraction functions (Figure 3e,f), indicating that the development of this cancer may involve the reversal of muscle cell differentiation found in the oral tongue. In addition, we have now identified specific genes (e.g, INHBA and WIF1), which while previously implicated in other cancer types, to our knowledge have not previously been implicated in carcinogenesis of OSCC.

Although deep sequencing, like microarray hybridization, is well suited to the task of profiling gene expression across tissues and individuals, it is also capable of interrogating aspects of gene expression that have typically eluded microarrays (e.g., detecting the presence of novel, alternative splice forms [4]). Here we have studied allelic imbalance, another aspect of gene expression that is difficult to study by microarray. The detection of imbalanced expression of alleles in cancer samples is a new and potentially powerful way to gain insights into cancer biology. Deletions, amplifications, point mutations and changes in the *cis* regulation of genes should all be reflected in the relative expression of alleles. We have scanned each normal and tumor transcriptome and assessed at each position the relative frequencies of the nucleotides sequenced. We conclude there is widespread allelic imbalance between normal and tumor samples: 185 to 345 sites from 121 to 209 unique genes passed our very conservative criteria. Interestingly, the three patients studied share many more allelically imbalanced genes than expected by chance, but the specific sites at which AI was detected typically differ between patients. It is remarkable that the set of genes allelically imbalanced in more than one patient is enriched for the same cancer-related functions as the set of differentially expressed genes (compare Figure 4c to Figure 3e,f), despite the fact that most of the AI genes are not themselves differentially expressed (Figure 4d). This latter observation may indicate that perturbation of the most differentially expressed genes tends to occur through *trans* regulatory mechanisms affecting both alleles. It also suggests that among the vast number of genes with changed expression in a tumor, some of the most causally relevant are those that change very little. For example, it is easy to imagine how a small perturbation to a central signaling protein may have large downstream effects. The observation that these genes are frequently perturbed in other cancers further argues for the relevancy of the AI genes. Overall, these results suggest that allelic imbalance may play a broad role in cancer biology and that its detection may provide a fruitful and novel avenue towards the discovery of new cancer genes. The development of more sophisticated algorithms for detecting and classifying AI along with the integration of complementary genotypic data, should allow greater insights into the mechanisms underlying AI and thus the genetic and epigenetic bases for development of various cancers. However, even with our rudimentary approach we have already observed changes that deserve further study, such as the allelic imbalance of DST and the possible imprinting switch at the IGF2/H19 locus (Figure 5). The possibility that imprinting was not only lost, but actually reset in development of this cancer, is intriguing and analogous to the phenomenon of epigenetic reprogramming that occurs during germ line development [69].

Finally, the combination of transcriptome sequencing and genome sequencing affords the opportunity to characterize the genomic mutations underlying alterations in gene expression in a tumor. We sequenced the tumor and normal genomes of one patient and used these data to determine copy number changes (CNCs) between the two samples. Highly concordant results were obtained by other methodologies (qPCR and aCGH; Figure S2), thereby validating the use of deep sequencing to measure CNC in primary tumors. We directly compared the CNCs identified in this tumor to changes in the tumor's transcriptome across the same genomic regions and thereby observed a strong correlation between changes in copy number and changes in gene expression (Figure 6a,b,c), an association that has been observed for other cancers in the past [70], [71]. Thus, much of the up- and down-regulation of genes observed in this tumor is likely to be driven by direct amplifications and deletions of the genomic regions containing these mis-regulated genes. Consistent with this model and the idea that deletions and amplifications in the tumor will tend to be allele-specific, we observe that the expression of heterozygous SNPs falling within CNV regions is significantly more imbalanced than the expression of heterozygous SNPs outside of CNV regions (Figure 6d). Although the number of samples examined here is low and thus the wider biological conclusions only preliminary, we believe the experimental and analytical methods developed and combined here offer a first glimpse at the power of pairing genomic and transcriptomic sequencing to understand the genetic basis of cancer development.

## Materials and Methods

### Ethics Statement

This study was conducted according to the principles expressed in the Declaration of Helsinki. The study was approved by the Institutional Review Board of the Mayo Clinic. All patients provided written informed consent for the collection of samples and subsequent analysis.

### Collection and Processing of Tissues

All tissues used in this study were collected from patients undergoing treatment at Mayo Clinic, Rochester, MN. Tumor samples were obtained from patients undergoing surgical treatment for oral squamous cell carcinoma (OSSC). Normal samples were collected from the negative surgical margins. Following surgical excision, a portion of the tissue was immediately processed and snap-frozen in liquid nitrogen for storage and future use. The remainder was processed for clinical examination and long-term storage in the tissue archives of Mayo Clinic, Rochester MN, according to standard clinical protocols. All patients consented to the use of their tissue for research and this study was approved by the Mayo Clinic Institutional Review Board. Each collected sample was frozen sectioned, changing the blade between samples, and mounted on positively charged glass slides that were stained with haematoxylin and eosin (H&E) by the Mayo Tissue and Cell Molecular Analysis Core facility. These slides were then evaluated by a qualified Mayo Pathologist (J. Lewis) to confirm the presence or absence of tumor in each sample. Appropriate tumor was circled and extreme care was taken to obtain only tumor-containing sections for subsequent isolation of DNA or RNA.

### DNA and RNA Extraction

Frozen tissues were compared to corresponding H&E slides following evaluation by pathology to verify classification of tumor or normal tissue status. Portions of tissue were removed for nucleic acid extraction, using disposable scalpels, in quantities <30mg. DNA was extracted from frozen tissue using the Invitrogen PureLink Genomic DNA Mini kit (Carlsbad, CA) according to the manufacturer's protocol. Total RNA was extracted from portions of the frozen tissue samples using the Qiagen RNAeasy Plus Kit (Valencia, CA) according to the manufacturer's protocol. Isolated DNA and RNA were quantified by NanoDrop ND1000 (ThermoFisher Scientific, Waltham, MA). RNA samples were further assessed for quality using the Agilent 2100 Bioanalyzer (Santa Clara, CA) prior to library construction.

### RNA Library Preparation

To construct libraries suitable for SOLiD™ System sequencing, 5 ug of total RNA was depleted of 18S and 28S rRNA using GLOBINclear™ (Ambion) buffers and reagents supplemented with biotinylated capture probes designed against these rRNAs and following the given protocol. The rRNA depleted samples (∼1 ug) were then fragmented by incubation with 1 unit of RNase III (Ambion) for 10 minutes in a 10 ul reaction volume containing 1X RNase III buffer supplied with the enzyme. The samples were then mixed with formamide gel loading dye and denatured for 10 min at 95°C and then separated on a flashPAGE™ gel apparatus using a modified procedure. The flashPAGE™ gel was first run for 15 minutes as per the given procedure and conditions. The lower running buffer was then removed and the lower chamber rinsed with nuclease-free water 2 times. The lower chamber was then replenished with fresh buffer and the gel was run for an additional 45 minutes. The lower running buffer was then removed and the RNA was purified using the flashPAGE™ clean up kit, producing RNA fragments ranging from ∼50–150 nt in size. This RNA was then used with the SOLiD Small RNA Expression Kit (Ambion) as per the given protocol, except the size range of products purified from the 6% native PAGE step was ∼140–200 bp in size. The final purified products were quantitated using a nanodrop and the size range of the products was confirmed by bioanalyzer analysis. The samples were then diluted and used for emulsion PCR.

### DNA Library Preparation

10 ug of each DNA sample was used to generate mate-paired libraries with a 2.5 kb insert size using standard manufacturer protocols. Briefly, DNA was sheared to a target size of 2.5 kb using a HydroShear® (Genomic Solutions). The resulting fragments were end repaired using the End-It™ (Epicentre) kit, methylated to protect EcoP151 sites, and ligated to CAP adapters. The DNA was then size selected by electrophoresis on a 1% agarose gel, and a band 2 kb to 3 kb was excised from the gel using a scalpel blade. DNA was recovered from the gel using QIAquick Gel Extraction Kit (Qiagen). The resulting DNA fragments were circularized by ligation to an internal adapter, and 25–27 bp ‘mates’ created by digestion with the type III restriction enzyme EcoP151. Double stranded P1 and P2 sequencing adapters were then ligated to the library and nick translated before final amplification using 14–15 cycles of PCR. Libraries were again purified by electrophoresis on a 3% agarose gel, excising the appropriate library band and recovering the DNA using the QIAquick Gel Extraction Kit (Qiagen). The size, quantity and quality of the resulting libraries were confirmed by analysis on a 2100 Bioanalyzer (Agilent) using a DNA 1000 chip before the library was diluted and used for emulsion PCR.

### Emulsion PCR

Templated beads were generated for sequencing using standard manufacturers' protocols. Briefly, an aqueous phase was prepared from the SOLiD ePCR kit containing AmpliTaq Gold DNA Polymerase UP, buffer, MgCl_2_, dNTP's, amplification primers and library template. The aqueous phase was then introduced to a whirling oil phase in an ULTRA-TURRAX® Turbo Drive (IKA) to create a water-in-oil emulsion. The emulsion was then transferred to a 96 well plate and thermocycled using the recommended PCR conditions. After PCR amplification, emulsions were broken using butanol, and the beads were washed, enriched, and terminal transferased before quantification and deposition onto a slide for sequencing.

### Whole Transcriptome Sequencing

Templated beads were deposited onto one full slide per sample. Massively parallel ligation sequencing was carried out to 50 bases using Applied Biosystems SOLiD System (V3 chemistry) and following the manufacturer's instructions.

### Mate-Pair Genome Sequencing

Templated beads for the normal and tumor samples were deposited across two sequencing slides, three quadrants per sample. Both forward and reverse tags from the mate-paired libraries were sequenced to 25 bases, using Applied Biosystems SOLiD System (V3 chemistry) and following the manufacturer's instructions.

### Alignment of Transcriptome Fragment Reads

Whole transcriptome reads were aligned using AB's SOLiD Whole Transcriptome Pipeline [72]. This software is open-source and freely available (http://solidsoftwaretools.com/gf/project/transcriptome/). An overview of the alignment strategy is presented in Figure S2. In all the analyses of gene expression presented here, only uniquely aligned reads were considered. A “uniquely aligned” read is defined as a read with a max scoring alignment to the genome scoring (1) at least 24 and (2) at least four higher than any of the other alignments of that read to the genome. Exon locations described in the text were taken from the alignments of RefSeq transcripts to version 18 of the human genome sequence (hg18), and are available at the UCSC Genome Browser website (http://genome.ucsc.edu/). All sequence data has been deposited at the MIAME compliant Gene Expression Omnibus (GEO) database at National Center for Biotechnology Information (http://www.ncbi.nlm.nih.gov/geo) and is accessible through accession number GSE20116.

### Alignment of Genomic Mate-Pair Reads

Mapping and pairing were performed with Applied Biosystems' alignment and pairing package (corona_lite, http://solidsoftwaretools.com/gf/project/corona/). The AB SOLiD alignment tool, mapreads, translates the reference sequence to dibase encoding (color space) and aligns reads in color space. The program guarantees finding all alignments between a read and the reference sequence with up to M mismatches (a user specified parameter, which was set to two here). Mapreads uses multiple spaced seeds (discontinuous word patterns) to achieve a rapid running time (Zhang et al., in preparation). Reads that align to the color space reference in only one location with up to two mismatches are referred to as “uniquely aligned”. Mate-pair reads were aligned individually and subsequently a pairing process was conducted to pair the individual reads. Pairing rescue was also performed, which uses one aligned tag as an anchor and searches for the other tag in a nearby window (of 1.4–3.7 kb, either upstream or downstream, depending on orientation of the anchored tag) with more relaxed criteria.

### Quantification of RefSeq Transcripts from RNA-Seq Data

For each of the 18,095 RefSeq transcripts, reads uniquely aligned within its genome-mapped exons were summed. One pseudo-count was added to this sum and the resulting modified raw transcript count was divided by the total number of uniquely aligned reads for the sample, yielding normalized transcript counts for each RefSeq transcript in each sample. Normalized transcript counts and TvN fold-changes can be found in Table S1. Because it is not yet clear whether genes expressed at the very lowest levels in a tissue are accurately measured by these methods, we applied a conservative raw count filter throughout our analysis of the RNA-Seq data, requiring that a transcript have at least fifty uniquely aligned reads in at least one tissue (tumor or normal) in all three patients. We did not normalize for transcript length because all further analyses focused on fold-changes between two conditions.

### Defining the Sets of Genes Mis-Regulated Across the Patients

To isolate the set of genes commonly mis-regulated in the development of OSCC, we rank ordered the TvN fold-changes for each patient. We then ranked transcripts by their median TvN rank across patients, considering further only the three hundred highest and three hundred lowest ranking genes as the sets of genes commonly up-regulated and down-regulated in OSCC development, respectively. Gene sets can be found in Table S3. The three hundred most up-regulated and down-regulated genes have median fold-change cutoffs of at least 3-fold, though typically the median fold changes are much higher, with a mean of 6-fold for the up-regulated genes and 33-fold for down-regulated set. A simulation, in which the pairing of transcripts and expression levels is randomly shuffled, indicates that less than 10% of our down-regulated and 20% of our up-regulated genes would have as extreme a median rank if there was no similarity in TvN expression profiles between patients (not shown). Thus, we estimate that these sets contain relatively few false positive genes, which are not truly differentially expressed across these three patients.

Although rank ordering was our original approach, we have also employed a recently proposed likelihood ratio test combined with a fold-change cutoff to define sets of mis-regulated genes [20] (Table S1). Using this statistical test (specifically, an FDR cutoff of 10^−4^ and 4-fold change requirement) results in very similar lists of up- and down-regulated genes. We performed GO analysis on the sets of up- and down-regulated genes defined in this manner and found that the resulting enriched biological functions are also very similar to before, changing none of our conclusions.

### Gene Expression Validation by Microarray

mRNA samples were reverse transcribed by priming off the polyA tail, producing cDNA pools. Each cDNA pool was amplified by *in vitro* transcription, generating biotin-labeled cRNA that was then hybridized to an Illumina HumanHT-12 v3 BeadChip array. Arrays were subsequently stained with Streptavidin-Cy3 and scanned with a high resolution Illumina scanner to determine fluorescence intensities. The raw intensity files from the arrays were pre-processed using Illumina's BeadStudio software for background subtraction and quantile normalization, producing normalized intensity values for each gene. Differential gene expression values, between the tumor and normal tissues of a particular patient, were compared with the corresponding values from RNA-Seq data (Table S2). The differential expression of a gene was calculated by applying a log_2_ transform to the ratio of modified, normalized intensities in the tumor and normal samples of a patient. The modified, normalized intensity for a gene was derived from its normalized intensity by subtracting the minimum normalized signal intensity across all genes in the sample and adding a value of one (to avoid dividing by zero). All microarray data is MIAME compliant and the raw data has been deposited at the MIAME compliant Gene Expression Omnibus (GEO) database at National Center for Biotechnology Information (http://www.ncbi.nlm.nih.gov/geo) and are accessible through accession number GSE19089.

### Gene Expression Validation by Reverse Transcription Quantitative PCR (RT-qPCR)

cDNA was produced using 2 ug of isolated RNA and the High Capacity cDNA Reverse Transcription Kit with RNase Inhibitor (P/N 4374966, Applied Biosystems, Foster City, CA). A total of 250 ng of the cDNA product was preamplified using the Applied Biosystems TaqMan® PreAmp Master Mix Kit according to standard protocol (P/N 4391128). Briefly, 250 ng of cDNA was amplified for 14 cycles with a pool of 20X TaqMan Gene Expression Assays specific to the target genes (P/Ns 4331182 and 4351372, Applied Biosystems). Following preamplification, the product was diluted 1:20 in 1 X TE buffer. The diluted preamplified cDNA was used in individual quantitative PCR reactions including TaqMan gene expression assays to measure the expression levels of target genes. To normalize the expression levels of each target gene, the cycle threshold (CT) of an abundantly-expressed control gene (GADPH, GUSB, PGK1, TBP; P/Ns 4333764F, 4333767F, 4333765F and 4333769F) was subtracted from the CT for each target gene of interest. This value was then used to calculate log gene expression changes across samples/conditions (ddCT).

### Allelic Imbalance Analysis and Validation

Allelic imbalance (AI) is a difference in the nucleotide frequencies at a given genomic position between two RNA samples. To quantify AI at each position in the genome, our method first tallies di-colors from reads aligned to that position that represent either a reference nucleotide or a single nucleotide substitution, filtering from further analysis invalid di-colors, which likely represent sequencing errors or more complex mutations. The nucleotide frequencies are then compared between samples and the significance of the AI is determined by applying a χ^2^ test of independence (on a 2×4 contingency table).

In our first attempt to investigate AI we only required that each genomic position have: (1) at least 15x coverage in the tumor and normal samples, (2) significant AI with χ^2^ *P*-value less than 10^−3^, and (3) more than a 10% change in absolute frequency of at least one allele. Using these criteria, our first round validation of 27 genomic positions by allele-specific PCR (as-PCR) yielded a 67% false positive rate (Figure S6), 72% of which could be attributed to false heterozygous genotypes (i.e., as-PCR of genomic DNA showed only one allele was present, whereas RNA-Seq found two). Upon careful examination we realized that two characteristics were prevalent amongst the false positives and not amongst the true positives. The first characteristic of false positives is that most have more than 90% of the reads supporting at least one nucleotide aligned to identical positions in the genome. In many of these cases a non-reference nucleotide was observed in only one of the two samples (normal or tumor) and that nucleotide was not present in dbSNP. We think these cases are likely attributable to errors introduced during reverse transcription that are subsequently amplified and over-sampled in later steps of the experimental protocol. Thus, in our second attempt to investigate allelic imbalance we added another criterion, requiring that at each genomic position, (4) less than 90% of the reads supporting each nucleotide can be aligned to identical positions in the genome. The second characteristic of false positives is that some tend to occur towards the end of alignments where there is some uncertainty about the pairing of read and genomic sequence. This mis-pairing can arise at a splice junction boundary when there is sequence similarity between the 5′ end of the intron and the 5′ end of the downstream exon. Thus in our second attempt to investigate allelic imbalance we conservatively discarded the last five colors of each read alignment. Employing this added criterion and filter reduced the false positive rate to 7%, while generating only one false negative (false negative rate is 4%) on the first round data. A second round of validation was also undertaken on a new set of 34 genomic positions, chosen prospectively with these revised criteria. For this second validation round we saw the false positive rate drop to 43%, only 27% of which could be attributed to false heterozygous genotypes from RNA-Seq. Thus these revised criteria have considerably improved our specificity and reduced artifacts likely arising from errors in reverse transcription that are subsequently over-sampled during sequencing.

### Allelic Imbalance Validation by Allele-Specific qPCR (as-qPCR) and Allele-Specific Reverse Transcription qPCR (as-RT-qPCR)

cDNA was produced by reverse transcription using the High Capacity Reverse Transcription Kit (P/N 4322171, Applied Biosystems) and manufacturer's instructions. as-qPCR and as-RT-qPCR were performed on an Applied Biosystems 7900HT Sequence Detection System. The 10 µl PCR mixture contains diluted RT products (for as-RT-qPCR) or 3 ng genomic DNA (for as-qPCR), 1x TaqMan® Genotyping Master Mixture (P/N 4371357, Applied Biosystems), 0.3 µM allele-specific forward primer, 0.2 µm TaqMan® probe, and 0.9 µm reverse primer. The reactions were incubated in 384-well plate at 95°C for 10 minutes, followed by 2 cycles of 95°C for 15 seconds and 58°C for 1 minute, and 48 cycles of 95°C for 15 seconds and 60°C for 1 minute. All allele-specific forward primers, TanMan® probes and reverse primers were manufactured at Applied Biosystems. For each genomic coordinate of interest, each allele was quantified (in each gDNA and cDNA sample) by obtaining CT values from at least two (and typically three) replicate reactions. The mean across reactions was computed and a dCT value calculated by subtracting the mean CT value for the second allele from the mean CT value for the first allele. Genomic coordinates with gDNA assays yielding absolute dCT values greater than 4.0 were deemed to be homozygous for the allele with lower CT value, while all others were assigned a heterozygous genotype (Figure S6a). Allelic imbalance of RNA between tumor and normal samples was estimated by calculating a ddCT value for each genomic coordinate assayed, by simply subtracting the dCT value of the normal cDNA sample from the corresponding dCT of the tumor cDNA sample. ddCT values were then compared to allelic imbalance measurements made by RNA-Seq (Figure S6).

### Analysis of Copy Number Variation (CNV)

The ratio of the number of uniquely aligned reads in paired tumor and normal samples for any given genomic sequence window is an estimate of the copy number change in the window. We modified Segseq [56], a recently developed CNV calling and segmentation algorithm that is based on this principle, to handle SOLiD system sequencing reads. The SegSeq implementation can not effectively handle the large volumes of data produced here due to memory limitations, so we divided our original datasets (∼50M reads per sample, ∼0.8x sequence coverage) into random subsets (∼12M reads each, ∼0.2x sequence coverage). We then applied the modified algorithm to each subset of the mate-pair reads from the normal and tumor samples of Patient 8. Reassuringly, the algorithm produced very similar results on the random subsets of data (not shown). Default parameters for Segseq were used: the size of local windows (-*W*) = *400*; the size of alignable windows (*-d, -e*) = 100 kb. The *P-*value cutoffs, *p_init_* and p_merge_, that control genome wide false positive CNV segments, were set such that we generated 1 false positive segment from about 20–30 false positive initial break points (see Chiang et al. [56] for details). Segments are listed in Table S6.

### Calculation of Differential Gene Expression Across Copy-Number Segments

To compare the CN changes observed between normal and tumor tissue to changes in gene expression, we calculated the differential expression of each genomic segment simply by summing the number of reads uniquely aligned to this region in the tumor sample and dividing it by the number of uniquely aligned reads to this region in the normal tissue sample. The resulting ratio was normalized by the ratio of the total reads uniquely aligned for each sample.

### Copy Number Validation by qPCR

TaqMan Copy Number (CN) Assays were performed according to the manufacturer's instructions (P/Ns 4400293 and 4400296, Applied Biosystems, Foster City, CA). In all, the copy numbers of 23 genes of interest were measured across 23 samples, derived from six normal tissues, 14 tumors, and two Coriell Institute for Medical Research (Camden, NJ) gDNA controls (see Table S7 for assay details). For each gene of interest, four replicate CT values were obtained per sample with FAM™-labeled and VIC®-labeled probes, assaying the gene of interest and a control gene, respectively. Both RNaseP and TERT were used as controls in separate assays (P/Ns 4403328 and 4403315, Applied Biosystems). Four dCT values were calculated by subtracting each VIC CT from the corresponding FAM CT. A single ddCT value, estimating the CN fold-change in a particular tumor sample, was calculated by subtracting the median of median dCTs of the six normal samples from the median dCT value for that particular tumor sample. The null hypothesis that the CN fold-change is zero was tested by a t-test in which variance was estimated by pooling the 24 dCT values from normal samples. This hypothesis was rejected and the CN fold-change deemed significant for p<0.05. Genes with significant CN fold-changes in two separate assays (one using the RNaseP locus as a control and the other using TERT) are highlighted in Figure S7.

### Copy Number Validation by Microarray (aCGH)

aCGH was performed using the Agilent Human Genome Microarray Kit 244K (Agilent Technologies, Santa Clara, CA) which contains ∼244,000 60-mer oligonucleotide probes spanning coding and non-coding genomic sequences with median spacing of 7.4 and 16.5 kb respectively. Arrays were analyzed using the Genepix 4200A scanner (Axon Instruments, Union City, CA) and the Agilent Feature Extraction software (v9.1). Copy number segments were obtained with the Agilent CGH Analytics software (v.3.4), using the ADM-1 algorithm and default settings [73].

## Supporting Information

Figure S1 Whole transcriptome (WT) experimental protocol. The protocol used to prepare total RNA for SOLiD sequencing is diagrammed above. This approach achieves strand-specificity by employing end-specific ligation of sequencing adapters to RNA, prior to the cDNA synthesis step. The P1 sequencing adapter is an RNA/DNA complex that contains a 6 bp 3′ single-strand DNA overhang allowing it to hybridize only to the 5′ end of an RNA fragment/molecule and, likewise, the P2 adapter will hybridize only to the 3′ end. The ligase used is engineered specifically to prefer the types of double-stranded substrates produced by these hybridizations, effectively making proper hybridization a prerequisite for efficient ligation. So, when cDNA is sequenced off the P1 adapter we expect the read sequence to represent the underlying RNA in the 5′->3′ orientation and thus, after alignment, we can work out the genomic strand from which the RNA originated. Also, because RNA is fragmented prior to cDNA synthesis, the protocol is less biased with respect to the positional origin of inserts within transcripts.
(0.98 MB TIF)

Figure S2 Whole transcriptome (WT) alignment strategy. WT sequencing reads were analyzed using Applied Biosystems whole transcriptome software tools (http://solidsoftwaretools.com/gf/project/transcriptome/). Briefly, the reads generated from each sample are aligned to the human genome (hg18, NCBI Build 36.1). Given the size of our 50-base reads relative to average exon length (150 bases), we anticipated that a substantial fraction of reads (up to one third) will cover a splice junction. Hence, these reads will not align contiguously to the genome and standard read mapping methods (e.g., MAQ) will fail. Making the assumption that at least half of each read sequence originates from a contiguous region of the genome, we circumvented this problem by splitting each read into two 25 base non-overlapping halves and then mapping each read split to the genome independently using Applied Biosystems' color mapping tool (http://solidsoftwaretools.com/gf/project/mapreads/). During this mapping phase we allowed up to two mismatches and removed reads that align to more than 10 locations. The mapping of each half was extended along the mapped genomic region using colors from the other half until a maximal score was reached (+1 for a match and −1 for a mismatch). In cases where the read splits aligned to the same genomic location (i.e., cases where the read likely originated from a segment of RNA that did not contain a splice junction), the results from the two halves were merged. Alignment locations were subsequently used to generate counts for annotated exons, transcripts, and genes, as well as genomic coverage plots (WIG files) that were displayed in the UCSC Genome Browser.
(7.97 MB TIF)

Figure S3 RNA degradation and rRNA removal. An aliquot (1 ml; ranging from ∼15–100 ng) of each of the indicated RNA samples was processed on an Agilent Bioanalzer using a standard RNA nano chip. A good quality RNA sample should primarily show two distinct products representing the 18S and 28S rRNAs and produce RIN values of ∼9 using the standard bioanalyzer conditions. While these two distinct products are visible in these samples a large number of additional products are observed migrating at various sizes, indicating that these samples are compromised by degradation to varying degrees. The N8, T8 and N33 samples showed the greatest amount of degradation (RIN values 3.2, 4.4 and 3, respectively) while T33, N51 and T51 demonstrated less degraded RNA (RIN values 5.9, 6 and 6.1, respectively). The degree of fragmentation has a negative impact on the level of rRNA that can be removed from the sample using biotinylated capture probes. Any RNA fragments that lie outside the regions covered by the capture probes will not be effectively removed and can be captured and sequenced. Therefore, degraded RNA samples are expected to produce a higher number of tags representing rRNA than high quality intact RNA samples.
(7.99 MB TIF)

Figure S4 Validation of SOLiD whole transcriptome analysis with other gene expression measurement platforms. (A) Comparison of log_2_ (Tumor/Normal) values measured by the BeadArray microarray and SOLiD sequencing platforms. Pearson correlations are shown between the platforms, both within and between patients. (B–D) For each patient, a scatterplot of log_2_ (Tumor/Normal) values as measured by the BeadArray microarray and SOLiD sequencing platforms is shown. Points are colored by transcript abundance (blue indicating low and red indicating high abundance; there are roughly 5000 genes in each bin), revealing greater discordance for genes with low expression. (E–F) Eight down-regulated and eight up-regulated genes with expression measurements that were discordant between the SOLiD and BeadArray platforms were chosen for validation with TaqMan gene expression assays. Displayed are scatterplot comparisons of log_2_ (Tumor/Normal) expression as measured by (E) SOLiD and TaqMan (ρ = 0.84), and (F) BeadArray and TaqMan (ρ = 0.71).
(9.79 MB TIF)

Figure S5 Overview of allelic imbalance analysis. The top row contains histograms of allelic ratios for genomic positions in patients 8, 33, and 51 (as labeled). For a given genomic coordinate, the “allelic ratio” is the log_2_ of the number of reads aligned across that position that indicate the reference nucleotide divided by the number of reads that indicate the first non-reference nucleotide in dbSNP. Thus we only concern ourselves here with the subset of genomic positions for which an allele is listed in dbSNP and to which at least 15 reads are aligned. As expected if alleles tend to be expressed at equal levels, we see a trimodal distribution of allelic ratios, representing the three possible genotypes: homozygous reference, heterozygous and homozygous non-reference. Allelic ratio distributions for normal tissue samples and tumors are shown in red and blue, respectively. The bottom row contains histograms of χ^2^ allelic imbalance P-values for genomic positions in patients 8, 33, and 51 (as labeled). The P-values indicate the extent of allelic imbalance at a transcriptomic position and are calculated in the following manner: First di-colors from reads aligned to that position that represent either a reference nucleotide or a single nucleotide substitution are tallied. Invalid di-colors, which likely represent sequencing errors or more complex mutations, are filtered from further analysis. The nucleotide frequencies are then compared between samples (normal and tumor) by applying a χ^2^ test of independence (on a 2×4 contingency table). The true and simulated χ^2^ allelic imbalance P-value distributions are shown in red and blue, respectively. The true distribution is shifted to the right relative to the simulated distribution, signifying that many transcriptomic positions differ in nucleotide frequencies between normal and tumor samples more than expected by sampling alone.
(1.65 MB TIF)

Figure S6 Validation of allelic imbalance. In our first round of validation, 27 transcriptomic positions identified by SOLiD sequencing to have AI were selected for genotyping and validation by allele-specific PCR (as-RT-qPCR). (A) The 27 positions and various associated statistics are listed, one position per row. The “p-value” indicates the significance of relative AI between conditions and is calculated from a χ^2^ test of independence on the read counts (Methods). A “read count” is simply the number of reads aligned across the transcriptomic position that indicate the given nucleotide. Because our fragment sequence read length is 50, it is theoretically possible that reads aligned across a given transcriptomic position could have as many as 50 “unique read start positions” in the transcriptome (due to alternative splicing, the number could actually be higher in some cases). The rows are ordered by validation success (first 9 rows represent validated positions and the last 18 rows represent positions that did not validate) and a clear pattern emerges: most of our false positives, but not our true positives, have one nucleotide in one biological sample that is supported only by reads with the same alignment start in the transcriptome. That particular nucleotide tends not to be present in dbSNP and is not present according to as-qPCR of genomic DNA. One scenario that would lead to this phenomenon is an error introduced during reverse transcription that subsequently gets amplified in library preparation and later over-sampled during sequencing. Please see the Methods section for our refined strategy, which reduces the false positive rate retrospectively from 67% to 7% on the first round validation data. N = normal and T = tumor. (B) On a second round validation of 32 additional sites chosen prospectively with the revised strategy the false positive rate dropped to 43%. The fraction of sites falsely ascribed heterozygous genotypes based on RNA-Seq dropped from 49% in the first round to 7% in the second round of valdiation. (C) A scatter plot of relative AI values produced by SOLiD sequencing (X-axis) and as-RT-qPCR (Y-axis). Here, we combine all transcriptomic positions assayed in the first and second rounds of validation that passed the revised criteria and had genotypes that were concordant between RNA-Seq and as-qPCR of genomic DNA (N = 37).
(3.96 MB TIF)

Figure S7 Validation of SOLiD CNV analysis with other CNV measurement platforms and across a panel of tumor samples. Comparison of SOLiD results to results from (A) TaqMan CNV assays and (B) Agilent 244K CNV microarrays. (A) There is strong concordance of copy number changes as measured by TaqMan and SOLiD across 23 assayed genes (ρ = 0.99). (B) Only high confidence results from the microarray platform (colored red) compare favorably with the SOLiD results (ρ = 0.97). Most of the low confidence microarray results (colored blue) are measured by a single probe, rather than multiple probes, on the array. (C) In addition to the tumor and matched normal samples of patient 8 (labeled “8_1”), the 23 TaqMan CNV assays (interrogating 23 genes of interest) were applied to a panel of 13 other tumor samples and a second section of normal/tumor tissue from patient 8 (labeled “8_2”). Two negative control gDNAs from Coriell were also assayed. Shaded in blue and yellow are genes with significant copy number decreases and increases, respectively (t-test; p-value<0.05 for two separate assays, using either the RNAseP or TERT loci as controls). Values listed are log_2_ (Tumor/Normal_median_). Matched normal tissues were only available for 5 of the 13 tumors, thus the median of the normal samples was used as the “normal sample” for each tumor/normal comparison and the variance for each t-test was estimated from the pool of normals. In general, the variance among normal samples is low; for patients where matched normal tissue is available, using the median normal rather than the true matched normal provides very similar measurements of copy number change.
(9.41 MB TIF)

Text S1 Coverage of annotated exons, transcripts and genes.
(0.03 MB DOC)

Table S1 Normalized transcript counts and differential (tumor versus normal) transcript expression values measured by RNA-Seq.
(7.66 MB XLS)

Table S2 Differential (tumor versus normal) gene expression values measured by microarray hybridization and RNA-Seq.
(5.96 MB XLS)

Table S3 The three hundred most up-regulated and down-regulated genes in a comparison of tumor to normal tissue across the three patients.
(0.04 MB XLS)

Table S4 Genes that are allelically imbalanced in tumor versus normal comparisons of at least one patient.
(0.05 MB XLS)

Table S5 Genes that are allelically imbalanced in tumor versus normal comparisons of at least two patients. Genomic positions examined and other details provided.
(0.09 MB PDF)

Table S6 Copy number changes of genomic segments in patient 8.
(0.05 MB XLS)

Table S7 Copy number assay details.
(0.02 MB XLS)
