# Supplementary figures and images for: Tumor Transcriptome Sequencing Reveals Allelic Expression Imbalances Associated with Copy Number Alterations
Source: PLoS One. 2010 Feb 19;5(2):e9317. doi: 10.1371/journal.pone.0009317 (PMC2824832; doi:10.1371/journal.pone.0009317)

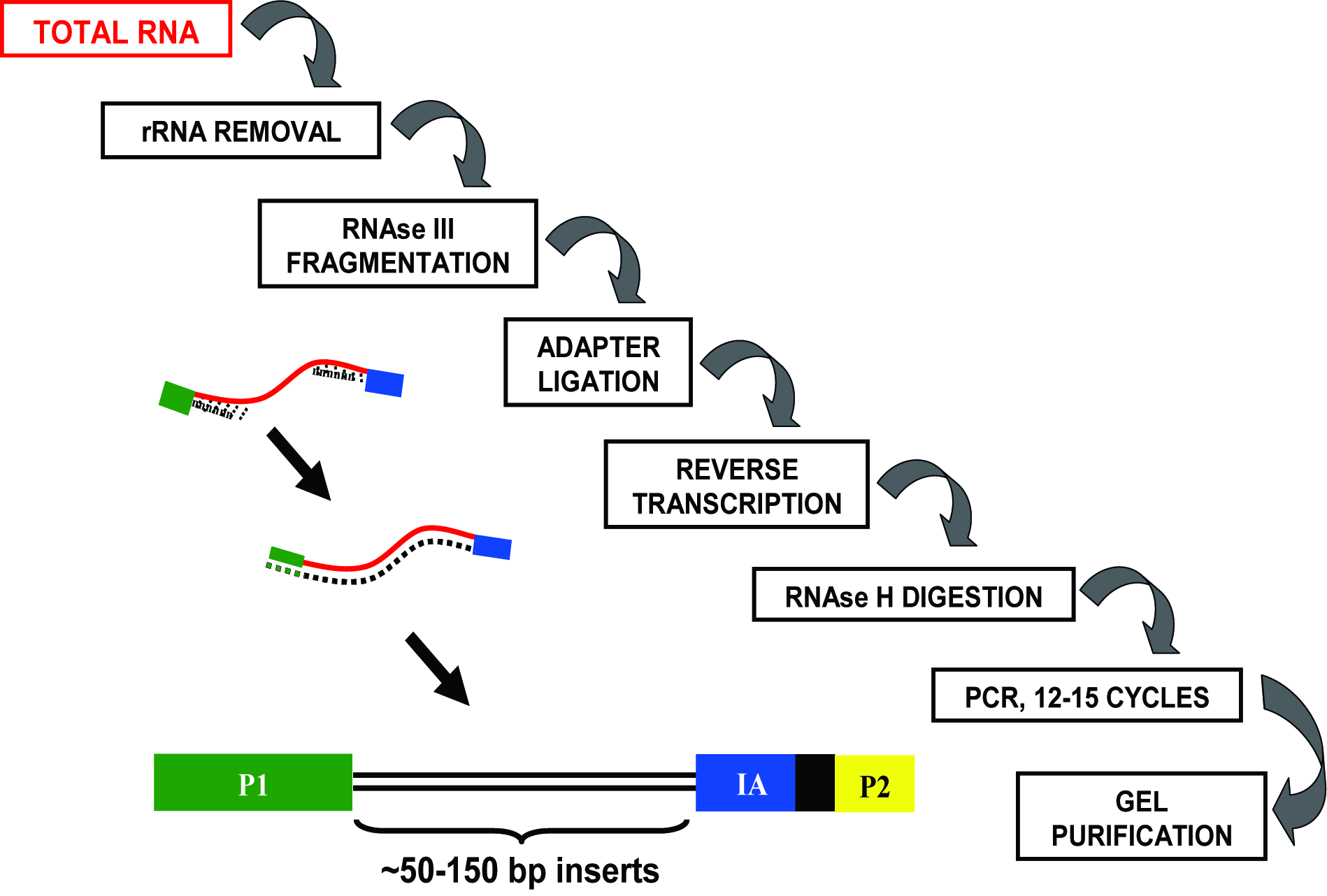

Supplement: Figure S1 — Whole transcriptome (WT) experimental protocol. The protocol used to prepare total RNA for SOLiD sequencing is diagrammed above. This approach achieves strand-specificity by employing end-specific ligation of sequencing adapters to RNA, prior to the cDNA synthesis step. The P1 sequencing adapter is an RNA/DNA complex that contains a 6 bp 3′ single-strand DNA overhang allowing it to hybridize only to the 5′ end of an RNA fragment/molecule and, likewise, the P2 adapter will hybridize only to the 3′ end. The ligase used is engineered specifically to prefer the types of double-stranded substrates produced by these hybridizations, effectively making proper hybridization a prerequisite for efficient ligation. So, when cDNA is sequenced off the P1 adapter we expect the read sequence to represent the underlying RNA in the 5′->3′ orientation and thus, after alignment, we can work out the genomic strand from which the RNA originated. Also, because RNA is fragmented prior to cDNA synthesis, the protocol is less biased with respect to the positional origin of inserts within transcripts. (0.98 MB TIF) [file pone.0009317.s001.tif]

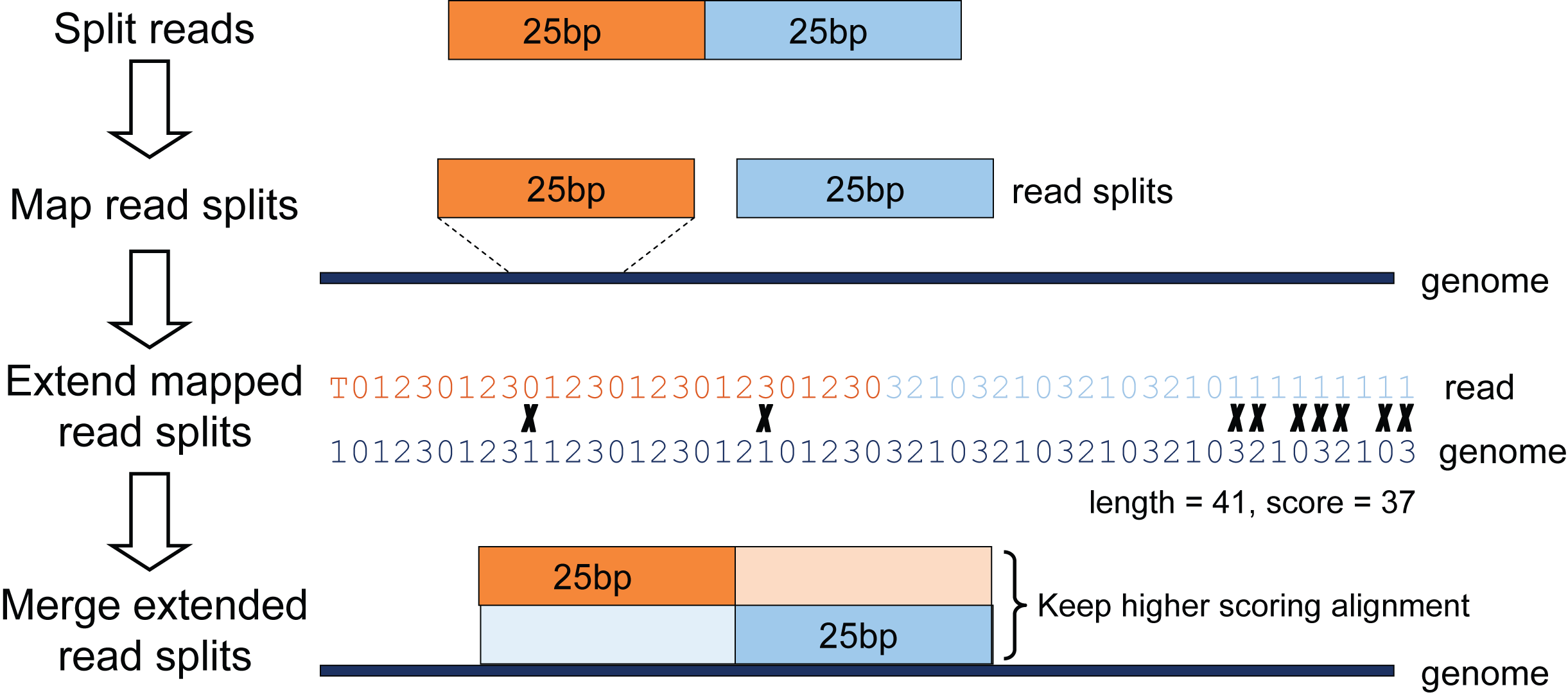

Supplement: Figure S2 — Whole transcriptome (WT) alignment strategy. WT sequencing reads were analyzed using Applied Biosystems whole transcriptome software tools (http://solidsoftwaretools.com/gf/project/transcriptome/). Briefly, the reads generated from each sample are aligned to the human genome (hg18, NCBI Build 36.1). Given the size of our 50-base reads relative to average exon length (150 bases), we anticipated that a substantial fraction of reads (up to one third) will cover a splice junction. Hence, these reads will not align contiguously to the genome and standard read mapping methods (e.g., MAQ) will fail. Making the assumption that at least half of each read sequence originates from a contiguous region of the genome, we circumvented this problem by splitting each read into two 25 base non-overlapping halves and then mapping each read split to the genome independently using Applied Biosystems' color mapping tool (http://solidsoftwaretools.com/gf/project/mapreads/). During this mapping phase we allowed up to two mismatches and removed reads that align to more than 10 locations. The mapping of each half was extended along the mapped genomic region using colors from the other half until a maximal score was reached (+1 for a match and −1 for a mismatch). In cases where the read splits aligned to the same genomic location (i.e., cases where the read likely originated from a segment of RNA that did not contain a splice junction), the results from the two halves were merged. Alignment locations were subsequently used to generate counts for annotated exons, transcripts, and genes, as well as genomic coverage plots (WIG files) that were displayed in the UCSC Genome Browser. (7.97 MB TIF) [file pone.0009317.s002.tif]

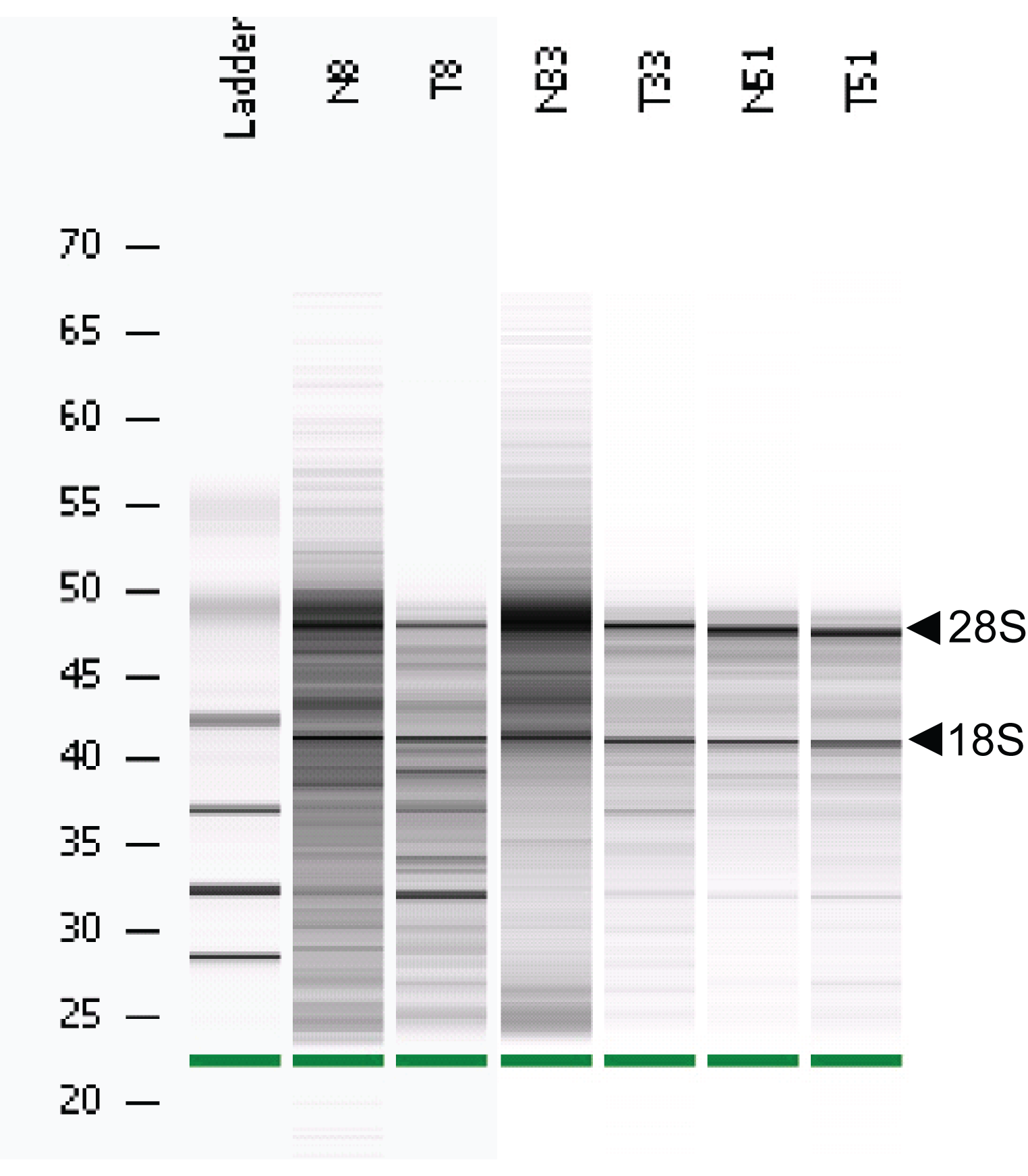

Supplement: Figure S3 — RNA degradation and rRNA removal. An aliquot (1 ml; ranging from ∼15–100 ng) of each of the indicated RNA samples was processed on an Agilent Bioanalzer using a standard RNA nano chip. A good quality RNA sample should primarily show two distinct products representing the 18S and 28S rRNAs and produce RIN values of ∼9 using the standard bioanalyzer conditions. While these two distinct products are visible in these samples a large number of additional products are observed migrating at various sizes, indicating that these samples are compromised by degradation to varying degrees. The N8, T8 and N33 samples showed the greatest amount of degradation (RIN values 3.2, 4.4 and 3, respectively) while T33, N51 and T51 demonstrated less degraded RNA (RIN values 5.9, 6 and 6.1, respectively). The degree of fragmentation has a negative impact on the level of rRNA that can be removed from the sample using biotinylated capture probes. Any RNA fragments that lie outside the regions covered by the capture probes will not be effectively removed and can be captured and sequenced. Therefore, degraded RNA samples are expected to produce a higher number of tags representing rRNA than high quality intact RNA samples. (7.99 MB TIF) [file pone.0009317.s003.tif]

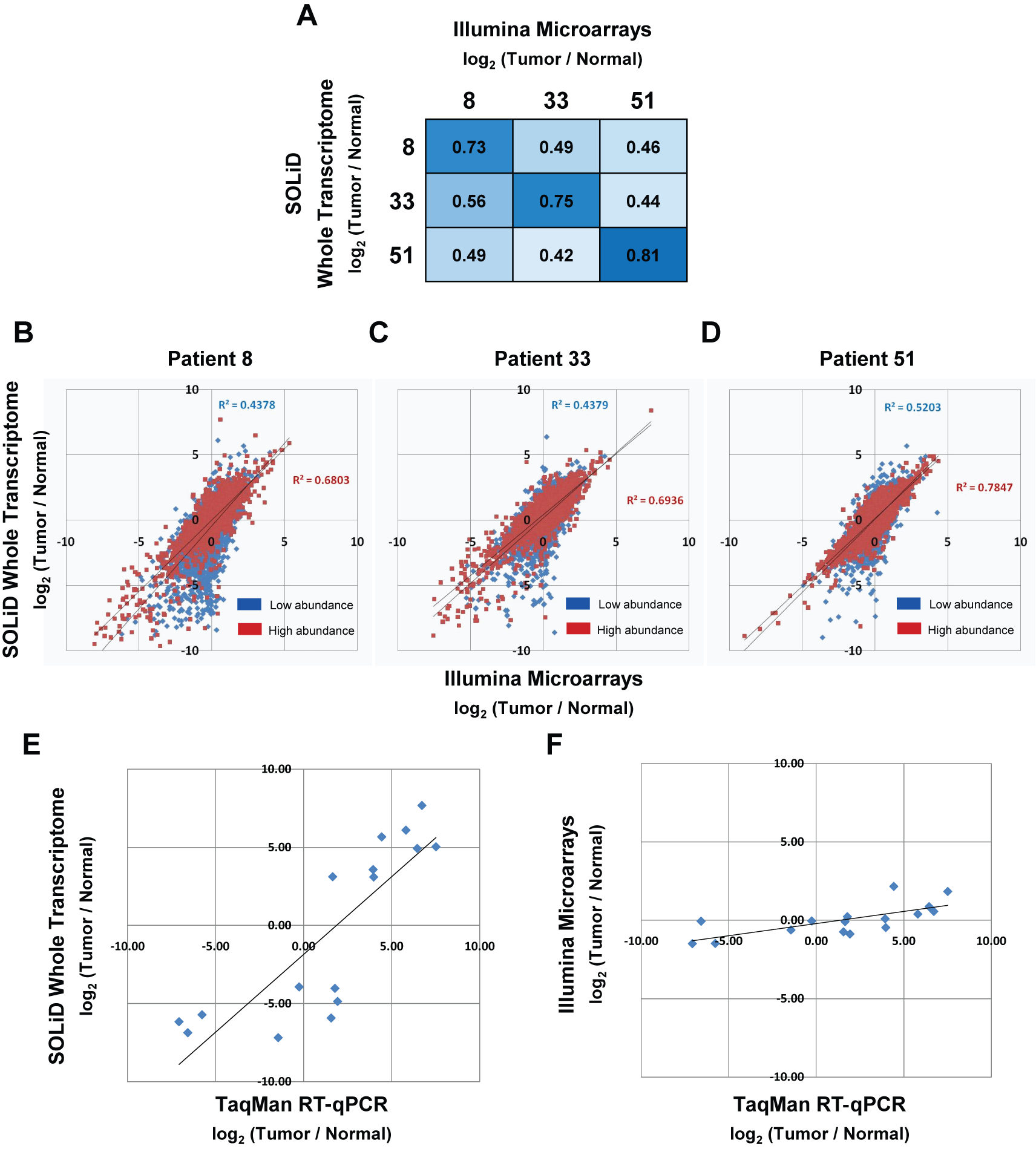

Supplement: Figure S4 — Validation of SOLiD whole transcriptome analysis with other gene expression measurement platforms. (A) Comparison of log2 (Tumor/Normal) values measured by the BeadArray microarray and SOLiD sequencing platforms. Pearson correlations are shown between the platforms, both within and between patients. (B–D) For each patient, a scatterplot of log2 (Tumor/Normal) values as measured by the BeadArray microarray and SOLiD sequencing platforms is shown. Points are colored by transcript abundance (blue indicating low and red indicating high abundance; there are roughly 5000 genes in each bin), revealing greater discordance for genes with low expression. (E–F) Eight down-regulated and eight up-regulated genes with expression measurements that were discordant between the SOLiD and BeadArray platforms were chosen for validation with TaqMan gene expression assays. Displayed are scatterplot comparisons of log2 (Tumor/Normal) expression as measured by (E) SOLiD and TaqMan (ρ = 0.84), and (F) BeadArray and TaqMan (ρ = 0.71). (9.79 MB TIF) [file pone.0009317.s004.tif]

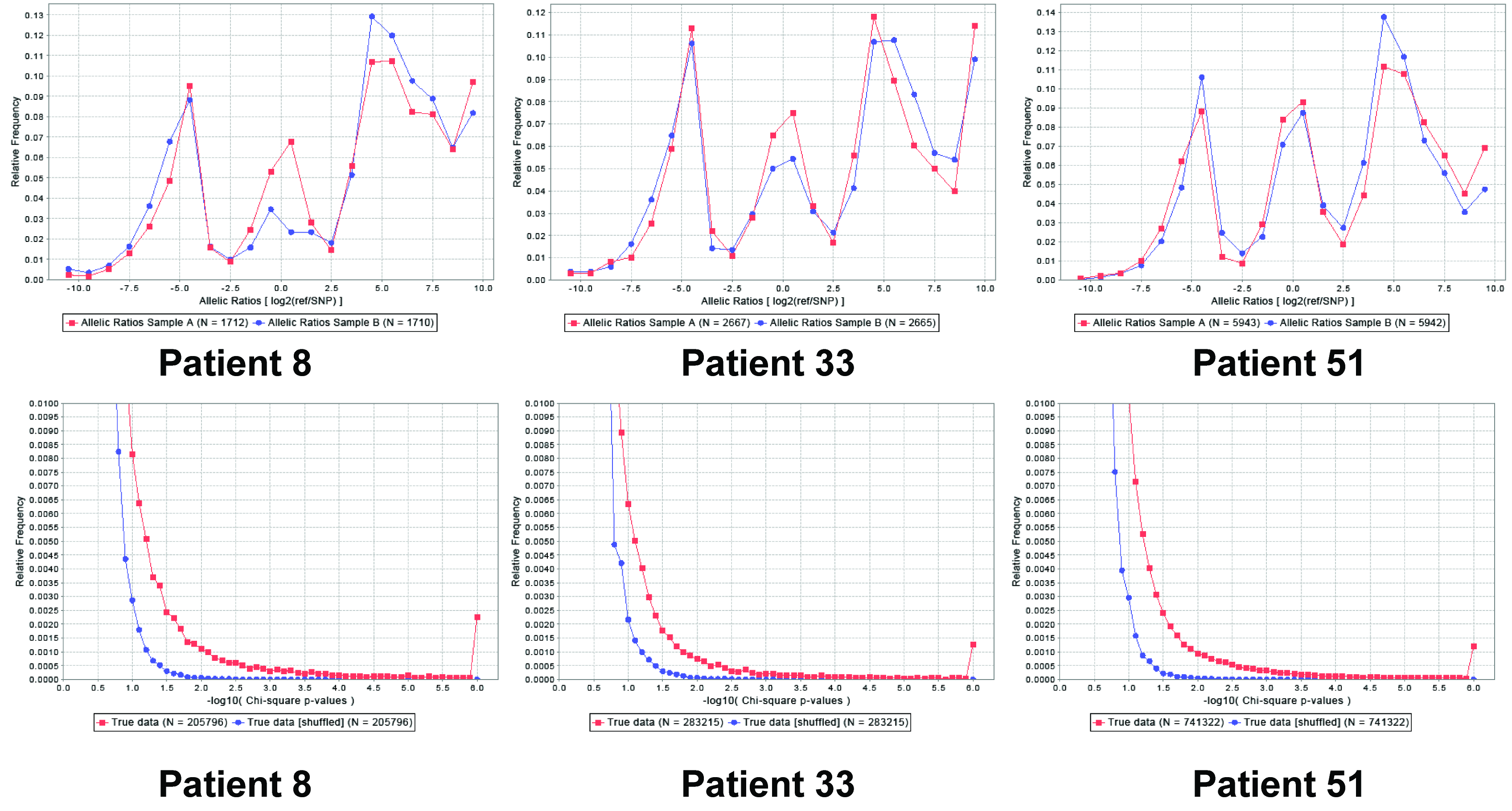

Supplement: Figure S5 — Overview of allelic imbalance analysis. The top row contains histograms of allelic ratios for genomic positions in patients 8, 33, and 51 (as labeled). For a given genomic coordinate, the “allelic ratio” is the log2 of the number of reads aligned across that position that indicate the reference nucleotide divided by the number of reads that indicate the first non-reference nucleotide in dbSNP. Thus we only concern ourselves here with the subset of genomic positions for which an allele is listed in dbSNP and to which at least 15 reads are aligned. As expected if alleles tend to be expressed at equal levels, we see a trimodal distribution of allelic ratios, representing the three possible genotypes: homozygous reference, heterozygous and homozygous non-reference. Allelic ratio distributions for normal tissue samples and tumors are shown in red and blue, respectively. The bottom row contains histograms of χ2 allelic imbalance P-values for genomic positions in patients 8, 33, and 51 (as labeled). The P-values indicate the extent of allelic imbalance at a transcriptomic position and are calculated in the following manner: First di-colors from reads aligned to that position that represent either a reference nucleotide or a single nucleotide substitution are tallied. Invalid di-colors, which likely represent sequencing errors or more complex mutations, are filtered from further analysis. The nucleotide frequencies are then compared between samples (normal and tumor) by applying a χ2 test of independence (on a 2×4 contingency table). The true and simulated χ2 allelic imbalance P-value distributions are shown in red and blue, respectively. The true distribution is shifted to the right relative to the simulated distribution, signifying that many transcriptomic positions differ in nucleotide frequencies between normal and tumor samples more than expected by sampling alone. (1.65 MB TIF) [file pone.0009317.s005.tif]

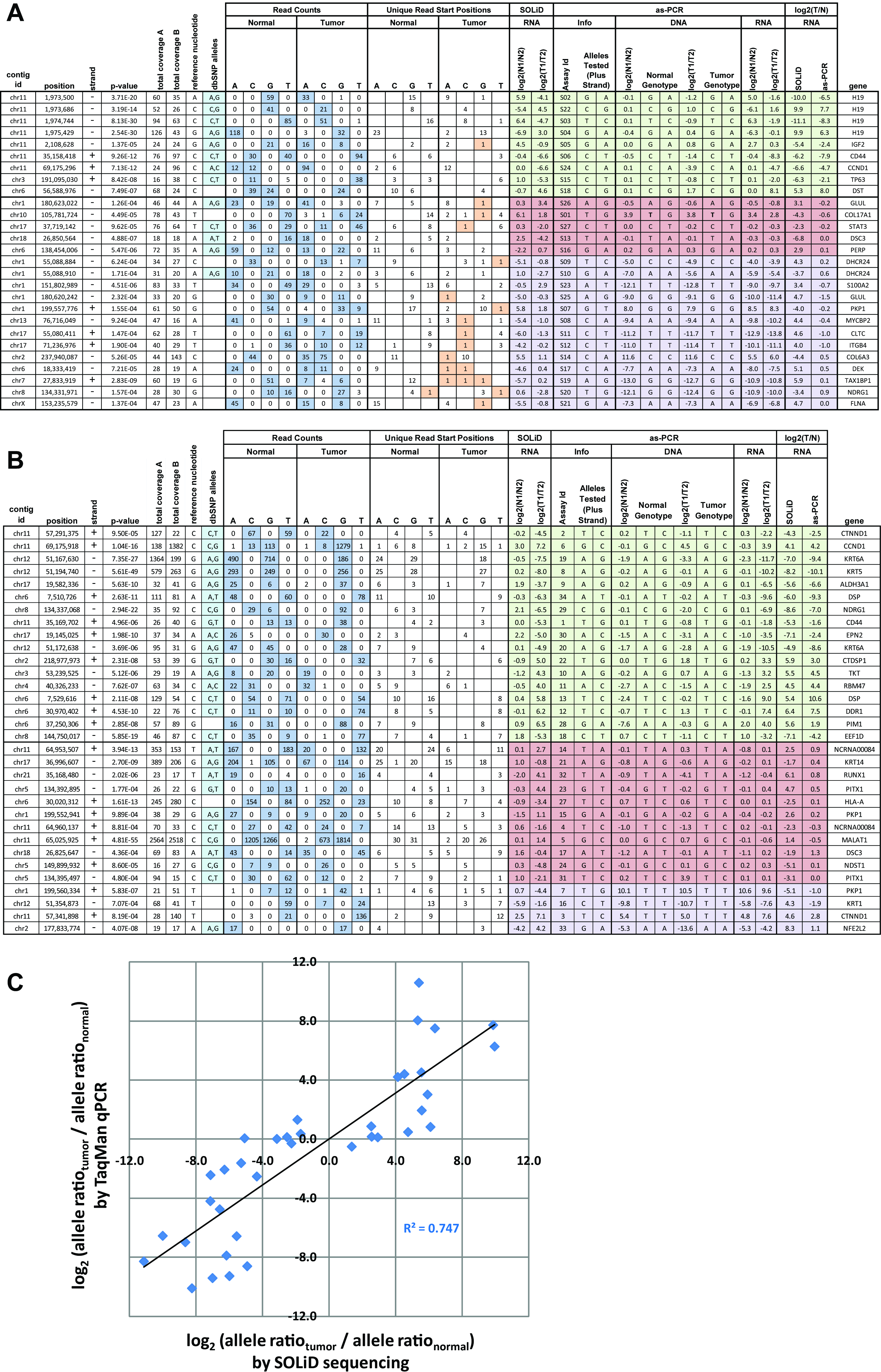

Supplement: Figure S6 — Validation of allelic imbalance. In our first round of validation, 27 transcriptomic positions identified by SOLiD sequencing to have AI were selected for genotyping and validation by allele-specific PCR (as-RT-qPCR). (A) The 27 positions and various associated statistics are listed, one position per row. The “p-value” indicates the significance of relative AI between conditions and is calculated from a χ2 test of independence on the read counts (Methods). A “read count” is simply the number of reads aligned across the transcriptomic position that indicate the given nucleotide. Because our fragment sequence read length is 50, it is theoretically possible that reads aligned across a given transcriptomic position could have as many as 50 “unique read start positions” in the transcriptome (due to alternative splicing, the number could actually be higher in some cases). The rows are ordered by validation success (first 9 rows represent validated positions and the last 18 rows represent positions that did not validate) and a clear pattern emerges: most of our false positives, but not our true positives, have one nucleotide in one biological sample that is supported only by reads with the same alignment start in the transcriptome. That particular nucleotide tends not to be present in dbSNP and is not present according to as-qPCR of genomic DNA. One scenario that would lead to this phenomenon is an error introduced during reverse transcription that subsequently gets amplified in library preparation and later over-sampled during sequencing. Please see the Methods section for our refined strategy, which reduces the false positive rate retrospectively from 67% to 7% on the first round validation data. N = normal and T = tumor. (B) On a second round validation of 32 additional sites chosen prospectively with the revised strategy the false positive rate dropped to 43%. The fraction of sites falsely ascribed heterozygous genotypes based on RNA-Seq dropped from 49% in the first r [file pone.0009317.s006.tif]

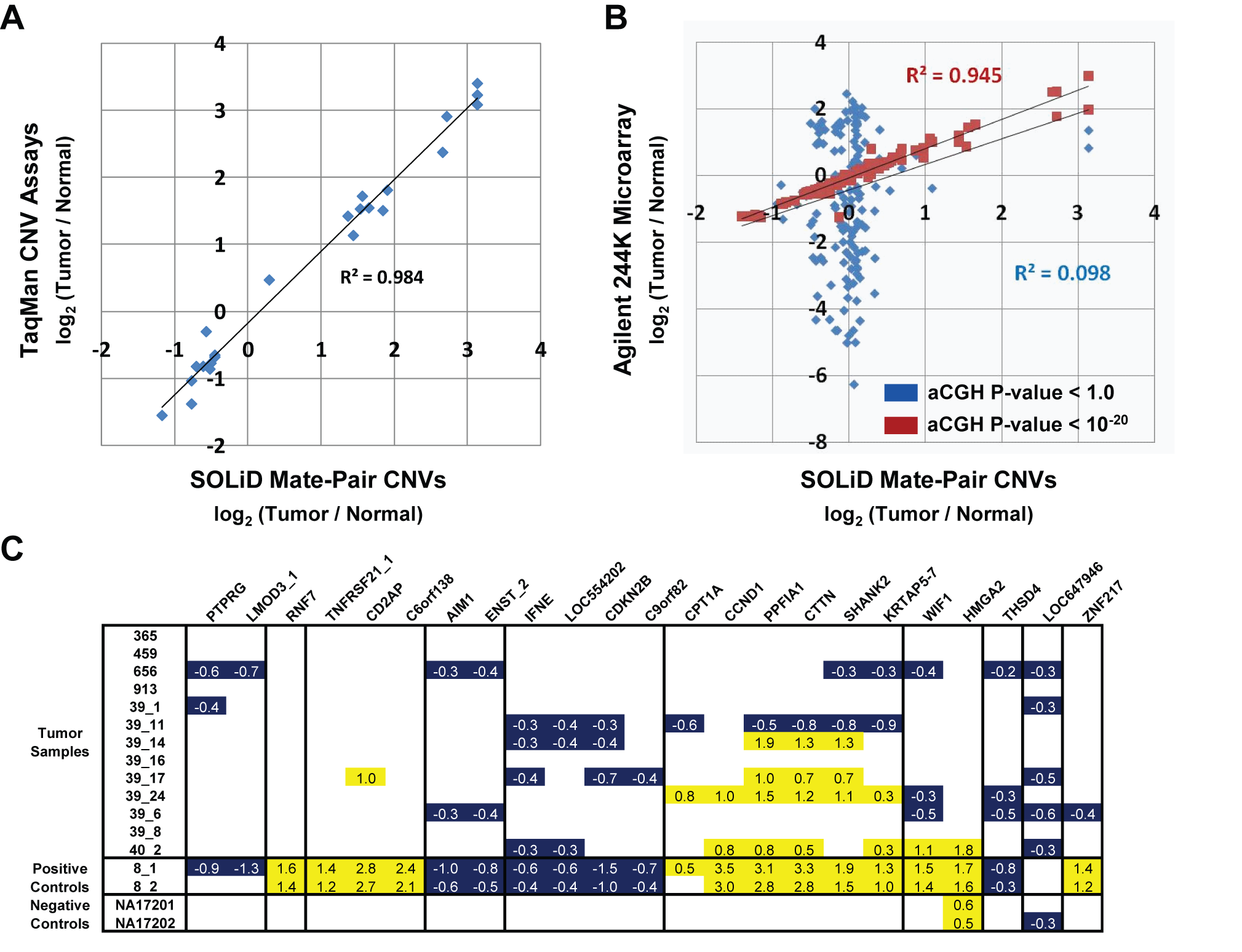

Supplement: Figure S7 — Validation of SOLiD CNV analysis with other CNV measurement platforms and across a panel of tumor samples. Comparison of SOLiD results to results from (A) TaqMan CNV assays and (B) Agilent 244K CNV microarrays. (A) There is strong concordance of copy number changes as measured by TaqMan and SOLiD across 23 assayed genes (ρ = 0.99). (B) Only high confidence results from the microarray platform (colored red) compare favorably with the SOLiD results (ρ = 0.97). Most of the low confidence microarray results (colored blue) are measured by a single probe, rather than multiple probes, on the array. (C) In addition to the tumor and matched normal samples of patient 8 (labeled “8_1”), the 23 TaqMan CNV assays (interrogating 23 genes of interest) were applied to a panel of 13 other tumor samples and a second section of normal/tumor tissue from patient 8 (labeled “8_2”). Two negative control gDNAs from Coriell were also assayed. Shaded in blue and yellow are genes with significant copy number decreases and increases, respectively (t-test; p-value<0.05 for two separate assays, using either the RNAseP or TERT loci as controls). Values listed are log2 (Tumor/Normalmedian). Matched normal tissues were only available for 5 of the 13 tumors, thus the median of the normal samples was used as the “normal sample” for each tumor/normal comparison and the variance for each t-test was estimated from the pool of normals. In general, the variance among normal samples is low; for patients where matched normal tissue is available, using the median normal rather than the true matched normal provides very similar measurements of copy number change. (9.41 MB TIF) [file pone.0009317.s007.tif]
