# Supplementary material for: Tumor Transcriptome Sequencing Reveals Allelic Expression Imbalances Associated with Copy Number Alterations
Source: PLoS One. 2010 Feb 19;5(2):e9317. doi: 10.1371/journal.pone.0009317 (PMC2824832; doi:10.1371/journal.pone.0009317)
Supplement: Text S1 — Coverage of annotated exons, transcripts and genes. (0.03 MB DOC) [file pone.0009317.s008.doc]

**Text S1. Coverage of annotated exons, transcripts and genes.**

At this sequencing depth we detect at least one read uniquely aligned to between 102,540 and 150,333 of the 195,148 total RefSeq-annotated exons per sample. With the more conservative requirement of at least two reads uniquely aligned, each with different alignment start positions, we detect between 80,905 and 136,674 of the RefSeq exons per sample. Despite the high levels of detection, 60-67% of uniquely mapped reads align outside of RefSeq exons. While some of these may be derived from novel non-coding transcripts, most of these reads align to regions that are intragenic to and on the same strand as RefSeq transcripts. Therefore, the majority is presumably derived from the intronic regions of pre-mRNAs present in the ribo-depleted total RNA used for library construction.
