# Supplementary material for: Tumor Transcriptome Sequencing Reveals Allelic Expression Imbalances Associated with Copy Number Alterations
Source: PLoS One. 2010 Feb 19;5(2):e9317. doi: 10.1371/journal.pone.0009317 (PMC2824832; doi:10.1371/journal.pone.0009317)
Supplement: Table S5 — Genes that are allelically imbalanced in tumor versus normal comparisons of at least two patients. Genomic positions examined and other details provided. (0.09 MB PDF) [file pone.0009317.s013.pdf]

	AI?			Gene Expression log2 (Tumor/Normal)			Allelic Imbalance (AI)												neighbor concord?	adhesion/ECM/lamina?		
							Patient 8			Patient 33			Patient 51									
	8	33	51	8	33	51	1 -> 2	2 -> 1	2 -> 2	1 -> 2	2 -> 1	2 -> 2	1 -> 2	2 -> 1	2 -> 2							
DST	●	●	●	-1.3	0.1	0.0		L->F		Syn, I/NSS			G->R			chr6	-	56,596,090	!	✔	dystonin isoform 1e precursor	member of the plakin protein family of adhesion junction plaque proteins. some isoforms are expressed in epithelial tissue, anchoring keratin-containing intermediate
PERP	●	●	●	0.7	0.0	1.7			3 (2)			Syn, R->P			3	chr6	-	138,461,949	✔	✔	PERP, TP53 apoptosis effector	downregulation of PERP correlated with metastatic capacity of human melanoma cell lines
FAT2	○	●	●	0.1	0.3	1.9						Syn		S->L, I	I->M, Syn	chr5	-	150,896,272	✔	✔	FAT tumor suppressor homolog 2	Drosophila and human homolog is a tumor suppressor gene
MALAT1	●	●	●	0.3	-0.6	0.3			NC			NC			NC	chr11	+	65,026,161	✔	✘	metastasis associated lung adenocarcinoma transcript 1	metastasis associated lung adenocarcinoma transcript 1 (non-protein coding) (MALAT1), non-coding RNA.
DSC3	●	●	●	0.6	0.5	1.1			3			3, K->R, S->T		3 (3)		chr18	-	26,850,414	✔	✔	desmocollin 3 isoform Dsc3a preproprotein	a calcium-dependent glycoprotein that is a member of the desmocollin subfamily. cell adhesion and desmosome formation.
CD44	●	●	●	-0.9	0.5	1.3		Syn, I				K->R			I/NSS	chr11	+	35,163,759	!	✔	Cd44 molecule	adhesion molecule involved in migration, cell fusion and resportion in osteoclasts that also plays a role in cellular metastasis.
GJB2	○	●	●	0.6	-0.7	1.2				W->C				I, 3		chr13	-	19,662,360	!	✔	gap junction protein, beta 2	forms gap junctions; may play a role in epididymal development
CTNND1	●	●	○	1.2	0.0	1.0		I, 3				I, 3				chr11	+	57,314,519	✔	✔	catenin, delta 1 isoform 1A	member of the Armadillo protein family, which function in adhesion between cells and signal transduction. Multiple translation initiation codons
CCND1	●	●	○	3.7	-0.1	1.2		3	3			3				chr11	+	69,171,738	!	✘	cyclin D1	regulatory subunit of CDK4 or CDK6; involved in regulation of the G1/S transition of the cell cycle
DSP	●	●	○	-0.1	-0.3	1.6		I->F, Syn				Syn				chr6	+	7,509,407	!	✔	desmoplakin isoform I	Desmosomes are intercellular junctions that tightly link adjacent cells. Desmoplakin is an obligate component of functional desmosomes that anchors intermediate fila plakophilin 1 and plakoglobin. The C-terminus of desmoplakin binds with intermediate filaments. In the mid-region of desmoplakin, a coiled-coiled rod domain is respo paraneoplastic pemphigus.
PKP1	●	○	●	0.0	0.6	1.5		3	I, I/NSS						I	chr1	+	199,543,971	!	✔	plakophilin 1	may be involved in molecular recruitment and stabilization during desmosome formation.
S100A2	●	○	●	0.1	0.7	1.3			I/NSS				I/NSS			chr1	-	151,802,569	✘	✘	S100 calcium binding protein A2	a member of the S100 family of proteins containing 2 EF-hand calcium-binding motifs. S100 proteins are involved in the regulation of cell cycle progression and differe
SKP1	○	●	●	0.2	-0.6	-0.4						5			5	chr5	-	133,530,302	!	✘	S-phase kinase-associated protein 1	component of SCF complexes, which are composed of this protein,**cullin** 1, a ring-box protein, and one member of the F-box family of proteins. This protein binds dire degradation by the proteosome. Specific F-box proteins recognize different target protein(s), and many specific SCF substrates have been identified including regulato
NCRNA00084	●	●	○	0.4	0.1	-0.3			NC (5)	NC		NC (3)				chr11	+	64,956,406	✔	✘	0	0
POLR2A	○	●	●	-0.5	1.2	0.4					Syn			3		chr17	+	7,343,614	✔	✘	polymerase (RNA) II (DNA directed) polypeptide	largest subunit of RNA polymerase II, the polymerase responsible for synthesizing messenger RNA in eukaryotes. The product of this gene contains a carboxy terminal phosphorylated in actively transcribing RNA polymerase. In addition, this subunit, in combination with several other polymerase subunits, forms the DNA binding dom
KRT14	●	○	●	-0.5	0.4	0.9			Syn, I/NSS						I/NSS	chr17	-	36,994,366	!	✘	keratin 14	member of the keratin family, the most diverse group of intermediate filaments. a type I keratin, is usually found as a heterotetramer with two keratin 5 molecules, a simplex. At least one pseudogene has been identified at 17p12-p11.
H19	●	●	○	-0.6	-2.1	-2.0	!!!		!!!		NC					chr11	-	1,974,311	!	✘	0	0
RUNX1	●	○	●	0.6	1.0	-0.4		I							I	chr21	-	35,212,716	✘	✘	runt-related transcription factor 1	putative transcription factor that may be involved in skeletal muscle gene regulation; human homolog is associated with a form of acute myeloid leukemia
KRT1	●	○	●	0.8	-3.0	1.5	3							Syn		chr12	-	51,357,622	✘	✘	keratin complex 2, basic, gene 1	--
MAP4	○	●	●	-1.5	-0.1	-1.0						3			3	chr3	-	47,897,033	!	✘	microtubule-associated protein 4	a major non-neuronal microtubule-associated protein. This protein contains a domain similar to the microtubule-binding domains of neuronal microtubule-associated interphase microtubule catastrophe promotion. Cyclin B was found to interact with this protein, which targets cell division cycle 2 (CDC2) kinase to microtubules. The
KRT6A	●	●	○	-1.8	-0.1	0.9		3 (2), I (3)				I, 3				chr12	-	51,170,336	✔	✘	keratin 6A	The protein encoded by this gene is a member of the keratin gene family. The type II cytokeratins consist of basic or neutral proteins which are arranged in pairs of he isoform.
HSPG2	○	●	●	0.1	0.4	-1.1						R->H			Syn	chr1	-	22,078,830	✘	✔	heparan sulfate proteoglycan 2	Heparan sulfate proteoglycan is a major component of basement membranes, where the molecule may be involved in the stabilization of other molecules as well as b
HNRNPH1	○	●	●	-0.1	-0.5	-0.3						I			I	chr5	-	178,978,556	✔	✘	heterogeneous nuclear ribonucleoprotein H1	--
SQSTM1	○	●	●	0.3	0.9	-0.7						Syn (2)			Syn (2)	chr5	+	179,189,065	✔	✘	sequestosome 1 isoform 1	interacts with PKC-zeta and GABA(C) receptors to form ternary postsynaptic complex in the retina
HLA-A	●	○	●	-0.3	0.3	0.5			Syn, F->L			3				chr6	+	30,019,971	!	✘	major histocompatibility complex, class I, A	HLA-A belongs to the HLA class I heavy chain paralogues. This class I molecule is a heterodimer consisting of a heavy chain and a light chain (beta-2 microglobulin). Cla
NDST1	●	○	●	-0.3	0.0	-0.7		Syn							Syn	chr5	+	149,892,916	!	✘	N-deacetylase/N-sulfotransferase (heparan	enzyme that catalyzes the transfer of sulfate from 3'-phosphoadenosine 5'-phosphosulfate to the nitrogen of glucosamine in heparan sulfate
EIF4G1	○	●	●	0.8	1.2	0.6						Syn	A->P	E->G		chr3	+	185,525,445	✘	✘	eukaryotic translation initiation factor 4	a component of the protein complex EIF4F, which is involved in the recognition of the mRNA cap, ATP-dependent unwinding of 5'-terminal secondary structure, and re
EPN2	●	●	○	-0.8	0.3	0.4		I				3				chr17	+	19,130,952	✔	✘	epsin 2 isoform b	may mediate membrane dynamics at the cell surface
PITX1	●	●	○	-0.9	-0.7	-2.8		Syn	I			Syn				chr5	-	134,394,593	✔	✘	paired-like homeodomain 1	paired-like homeobox transcription factor; transcription regulator of pituitary genes; genetic marker for nascent Rathke's pouch, precursor of anterior and intermedia
ALDH3A1	●	●	○	-0.9	1.4	-2.4		3	Syn			D->G				chr17	-	19,585,628	✔	✘	aldehyde dehydrogenase 3A1	aldehyde dehydrogenase that is expressed during hepatocarcinogenesis
CTSB	○	●	●	0.4	1.4	-0.5						3			3, L->V	chr8	-	11,750,249	!	✘	cathepsin B preproprotein	lysosomal peptidase
ACTG1	●	○	●	0.5	0.4	0.7			V->I						3	chr17	-	77,093,008	✘	✘	actin, gamma, cytoplasmic 1	Actins are highly conserved proteins that are involved in various types of cell motility, and maintenance of the cytoskeleton. The beta and gamma actins co-exist in m nonmuscle cells.
ZFP36L1	●	○	●	-0.5	-0.4	0.1			3						G->S	chr14	-	68,326,833	✘	✘	zinc finger protein 36, C3H type-like 1	putative nuclear transcription factor; member of the TIS11 family of early response genes; may regulate the response to growth factors [RGD].
ANXA1	○	●	●	-3.5	-2.2	-1.0						I/NSS			T->K	chr9	+	74,965,864	!	✘	annexin A1	plays a role in regulation of insulin secretion; may inhibit phopsholipase A2
SH3PXD2A	○	●	●	-0.2	0.6	0.4						3			Syn	chr10	-	105,474,464	!	✘	SH3 and PX domains 2A	--
PHKB	●	●	○	-0.2	0.3	0.4			I (2)			I				chr16	+	46,172,823	!	✘	phosphorylase kinase, beta	expression in regenerating muscle may be correlated with that of other phosphorylase kinase subunits
SRRM2	○	●	●	-0.4	0.9	-0.1						Syn	R->Q			chr16	+	2,751,872	!	✘	serine/arginine repetitive matrix 2	--
TMEM107	●	●	○	2.8	0.2	0.9			3		3					chr17	-	8,018,730	✔	✘	transmembrane protein 107 isoform 1	--
HLA-DRB6	○	●	●	-0.9	0.1	-1.2						NC			NC	chr6	-	32,632,112	!	✘	major histocompatibility complex, class II, DR beta 6	Homo sapiens major histocompatibility complex, class II, DR beta 6 (pseudogene) (HLA-DRB6), non-coding RNA.
ALKBH5	○	●	●	-0.9	1.6	0.4						3		3		chr17	+	18,040,792	✘	✘	alkB, alkylation repair homolog 5	--
BAT2L	○	●	●	-1.1	1.2	0.4						R->L			3	chr9	+	133,330,347		✘	HLA-B associated transcript 2-like	--
CDKN2AIPNL	○	●	●	1.2	-0.3	-0.2										chr5	-	133,610,065		✘	CDKN2A interacting protein N-terminal like	0
AHNAK	●	○	●	-0.2	0.0	0.5	Syn							A->E		chr11	-	62,014,250	✘	✘	AHNAK nucleoprotein isoform 3	--
COL1A1	●	○	●	4.5	1.1	-1.1									I/NSS	chr17	-	45,625,227		✘	alpha 1 type I collagen preproprotein	pro-alpha1 chains of type I collagen whose triple helix comprises two alpha1 chains and one alpha2 chain. Type I is a fibril-forming collagen found in most connective t syndrome type VIIA, Ehlers-Danlos syndrome Classical type, Caffey Disease and idiopathic osteoporosis. Reciprocal translocations between chromosomes 17 and 22, w protuberans, resulting from unregulated expression of the growth factor.
COL4A1	○	●	●	2.3	0.4	-1.5				A->T, Syn						chr13	-	109,678,404	!	✘	alpha 1 type IV collagen preproprotein	the major type IV alpha collagen chain of basement membranes. Like the other members of the type IV collagen gene family, this gene is organized in a head-to-head
FAM129B	●	●	○	-1.2	-0.2	-0.7						3, I				chr9	-	129,344,264	!	✘	hypothetical protein LOC362115	--
ITGB4	○	●	●	1.1	4.2	2.5						Syn (2)				chr17	+	71,248,932	!	✔	integrin beta 4	beta subunit of integrin alpha6/beta4 which is a cell surface receptor for laminin; involved in hemidesmosome formation.
KRT13	●	●	●	-7.2	-1.7	-6.4			G->A	I			Syn			chr17	-	36,913,075	!	✘	keratin 13	human homolog plays a role in epidermal development
KRT5	●	○	●	-0.5	-0.3	1.6		3, I (2), Syn (2)	S->G						I/NSS (2)	chr12	-	51,197,568	✔	✘	keratin 5	cytoskeletal intermediate filament protein; provides a cortical scaffold to primary spermatocytes
PID1	●	○	●	-1.4	-3.5	-3.6			I			I				chr2	-	229,720,617	✘	✘	phosphotyrosine interaction domain containing 1	0
PLEC1	●	●	●	1.7	1.7	1.0							Syn			chr8	-	145,076,101	✘		plectin 1	cytoskeletal cross-linking protein; interacts with all three major groups of cytoskeletal proteins, actin filaments, microtubules, and intermediate filaments
TGM3	●	●	○	-3.9	-2.3	-7.1	3			3						chr20	+	2,247,169	✘		transglutaminase 3 precursor	Transglutaminases are enzymes that catalyze the crosslinking of proteins by epsilon-gamma glutamyl lysine isopeptide bonds. involved the later stages of cell envelop
